# Bayesian Analysis for Inference of an Emerging Epidemic: Citrus Canker in Urban Landscapes

**DOI:** 10.1371/journal.pcbi.1003587

**Published:** 2014-04-24

**Authors:** Franco M. Neri, Alex R. Cook, Gavin J. Gibson, Tim R. Gottwald, Christopher A. Gilligan

**Affiliations:** 1 Department of Plant Sciences, University of Cambridge, Cambridge, United Kingdom; 2 Saw Swee Hock School of Public Health, National University of Singapore and National University Health System, Singapore; 3 Department of Statistics and Applied Probability, National University of Singapore, Singapore; 4 Program in Health Services and Systems Research, Duke-NUS Graduate Medical School Singapore, Singapore; 5 Communicable Disease Centre, Tan Tock Seng Hospital, Singapore; 6 Department of Actuarial Mathematics and Statistics and the Maxwell Institute for Mathematical Sciences, Heriot-Watt University, Edinburgh, United Kingdom; 7 U.S. Dept. of Agriculture, Agricultural Research Service, U.S. Horticultural Research Laboratory, Fort Pierce, Florida, United States of America; Pennsylvania State University, United States of America

## Abstract

Outbreaks of infectious diseases require a rapid response from policy makers. The choice of an adequate level of response relies upon available knowledge of the spatial and temporal parameters governing pathogen spread, affecting, amongst others, the predicted severity of the epidemic. Yet, when a new pathogen is introduced into an alien environment, such information is often lacking or of no use, and epidemiological parameters must be estimated from the first observations of the epidemic. This poses a challenge to epidemiologists: how quickly can the parameters of an emerging disease be estimated? How soon can the future progress of the epidemic be reliably predicted? We investigate these issues using a unique, spatially and temporally resolved dataset for the invasion of a plant disease, Asiatic citrus canker in urban Miami. We use epidemiological models, Bayesian Markov-chain Monte Carlo, and advanced spatial statistical methods to analyse rates and extent of spread of the disease. A rich and complex epidemic behaviour is revealed. The spatial scale of spread is approximately constant over time and can be estimated rapidly with great precision (although the evidence for long-range transmission is inconclusive). In contrast, the rate of infection is characterised by strong monthly fluctuations that we associate with extreme weather events. Uninformed predictions from the early stages of the epidemic, assuming complete ignorance of the future environmental drivers, fail because of the unpredictable variability of the infection rate. Conversely, predictions improve dramatically if we assume prior knowledge of either the main environmental trend, or the main environmental events. A contrast emerges between the high detail attained by modelling in the spatiotemporal *description* of the epidemic and the bottleneck imposed on epidemic *prediction* by the limits of meteorological predictability. We argue that identifying such bottlenecks will be a fundamental step in future modelling of weather-driven epidemics.

## Author Summary

We consider emerging epidemics, arising, e.g., when a new pathogen is introduced in a host population. In face of the new threat, crucial control measures have to be implemented quickly, yet prior knowledge of the parameters underlying pathogen spread and transmission is often missing. Predictive modelling can greatly help in informing decision making by estimating those parameters from early observations of the outbreak. The important questions are then: can a modeller characterise the disease “soon enough,” i.e., within a useful time frame, in order to enact the proper control measures? At what stage of the outbreak can the future epidemic progress be reliably predicted? We analyse an outbreak of citrus canker, a wind-spread bacterial disease of citrus, in urban Miami. The model succeeds in capturing the main epidemiological features of the disease, but we find contrasting answers. The spatial scale of disease spread can be identified quickly and accurately from early observations. However, the rate of spread is rapidly changing in time, driven mainly by rare thunderstorms with very short-time predictability, which frustrates epidemic prediction.

## Introduction

Emerging epidemics are of increasing topical interest [1]. These emerging diseases pose new threats to human health [2]–[6], livestock [7]–[11] and crop production [12]–[14], as well as wildlife populations [15]–[17] and natural plant communities [18]–[21]. Such epidemics occur most frequently when exotic pathogens are introduced into new environments or when novel strains arise that enable a pathogen to grow in a previously unfavourable environment [1].

One of the principal challenges in managing emerging epidemics is to predict the likely future development of disease in order to assess the severity of the invasion prior to instituting control measures. However, prediction is difficult when little is known about how a new pathogen is likely to continue to spread in an alien environment, and frequently the underlying epidemiological parameters that influence the spread of disease are not known. Even when there is prior knowledge of a pathogen, as for example foot and mouth epidemics in the UK in 1967, 1982 and 2001, different pathogen strains, changes in farming practices or environmental conditions can markedly change the extent and speed of disease spread through the landscape [7], [9], [22], [23]. Whereas, for example, the spread of foot and mouth disease in the 1967 epidemic was relatively localised, occurring mainly by aerial dispersal [24], changes in the frequency and distance of livestock movements over large distances [25] led to a strikingly, topologically different epidemic in 2001 [7], [9]. Numerous other examples have been reported of variability in epidemic outcome upon reintroductions of emerging pathogens. This is problematic, because rapid decisions about the introduction of disease control strategies often have to be made early in the course of an emerging epidemic. Sometimes, options are clear. Immediate control aimed at eradication is initiated as soon as an outbreak is detected for certain statutory diseases. Actions against the H1N1-2009 pandemic influenza worldwide [26], foot and mouth disease in the UK [8], and Asian soya bean rust in several US states [27] are good examples amongst others of human, livestock and crop diseases that attract an immediate response.

For other diseases, policy makers and disease control authorities may wish to wait to assess the likely severity of the infestation in order to consider the likely costs and benefits of control; delay may also be necessary to mobilise resources. Informed decision making invokes a series of questions about how to make inferences about the emerging epidemic: what type of epidemiological model can be used to characterise the epidemic and to predict future spread of disease? Where are the susceptible hosts and how are they distributed in the landscape? How is disease transmitted and what are the values of the epidemiological parameters for transmission and dispersal? How soon during the course of the epidemic can the parameters be reliably estimated? How should we take account of uncertainty? Here, we examine these questions using a combination of Bayesian statistical inference and a unique, spatially- and temporally-resolved data-set [28] for the invasion of a plant disease, Asiatic citrus canker, in Florida.

Asiatic citrus canker (ACC) is caused by the bacterium *Xanthomonas axonopodis* pv. *citri* (*Xac*). The pathogen can infect a very wide range of citrus and related hosts, causing defoliation, fruit blemishing and severe losses in quality and quantity of yield [29]. The pathogen is principally spread by wind-blown rain [29], [30]. It is not vector borne, other than by anthropomorphic transmission on machinery [29], but the spread is known to be exacerbated by leaf damage inflicted by the Asian leaf miner *Phyllocnistis citrella* that first appeared in Florida in 1993. There have been several independent introductions of *Xac* into Florida up until the mid 1990s [31]. The pathogen was originally introduced on imported seedlings from Japan in 1910 and declared eradicated, after extensive removal of infected and exposed susceptible trees, in 1933. An outbreak in Manatee county on the west coast of Florida was thought to have been eradicated in the 1980s, but ACC reoccurred within two years from surviving inoculum. A new infestation of ACC from a genetically different strain of *Xac* was reported in urban Miami on residential trees in 1995. The disease spread rapidly through Eastern and central Florida [29], triggering an extensive eradication programme, involving compulsory removal of ∼7M commercial, >4M nursery and 0.8M residential trees around infected sites, at a cost of >$1 billion. The eradication scheme was halted in 2006 following widespread dispersal of inoculum during several severe hurricanes in 2004 and the eventual determination that the disease had become endemic rendering eradication unattainable [32].

Here we focus on the early stage of the epidemic in urban Miami and, in particular, how to estimate the inherent spatial and temporal scales of the epidemic in order to predict the future course of an epidemic in a spatially heterogeneous urban setting. Infection on these trees constituted a potent source of inoculum that must be controlled were the disease threat to plantations to be economically managed. Accordingly, the USDA Agricultural Research Service initiated a detailed census of susceptible trees and the occurrence of ACC in five sites in Broward and Dade counties in the Miami region. The sites ranged from 2.6 km^2^ to 15.5 km^2^ [28]. The data provide a full census of susceptible trees, with 24 successive monthly snapshots for the occurrence of new infections. Retrospectively, the outcome of the epidemic at each site is known. Here we use subsets of the data at different stages of the epidemic to recreate different levels of ignorance about the future course of the epidemic. Then using Bayesian statistical inference and a stochastic model we compare model predictions with the known course of the epidemic. Specifically we ask:

- What is the appropriate epidemiological model to characterise the spread of disease?
- Is the epidemic self-contained at each site or is there evidence of ingress of inoculum from outside the site?
- How early in the epidemic can the epidemiological parameters be reliably estimated from disease snapshots?
- How does the starting time of observations affect the reliability of parameter estimates?
- Are the epidemiological parameters constant over time?
- Are the epidemiological parameters similar at each site?
- How do the predictions of the future evolution of the epidemic vary with the time of prediction and the amounts of data used for prediction?

By using the citrus canker outbreak to address these broad questions, we introduce and test methodologies that are applicable to a much broader class of spatially- and temporally-complex epidemics.

## Methods

The methods are organised as follows. The first three sections set the general problem by describing the data used for parameter estimation and the data collection process (first section), the models fitted to the data (second section), and methods for Bayesian parameter estimation (third section). Model selection methods are explained in the fourth section. In the fifth section, we discuss temporal-window techniques for the change of parameter estimates with time; the sixth section describes techniques for parameter changes amongst census sites. The seventh section describes goodness-of-fit tests. In the eighth and final section, we give details on simulating predictive distributions of epidemic outbreaks.

### Data for parameter estimation

**Figure 1:**
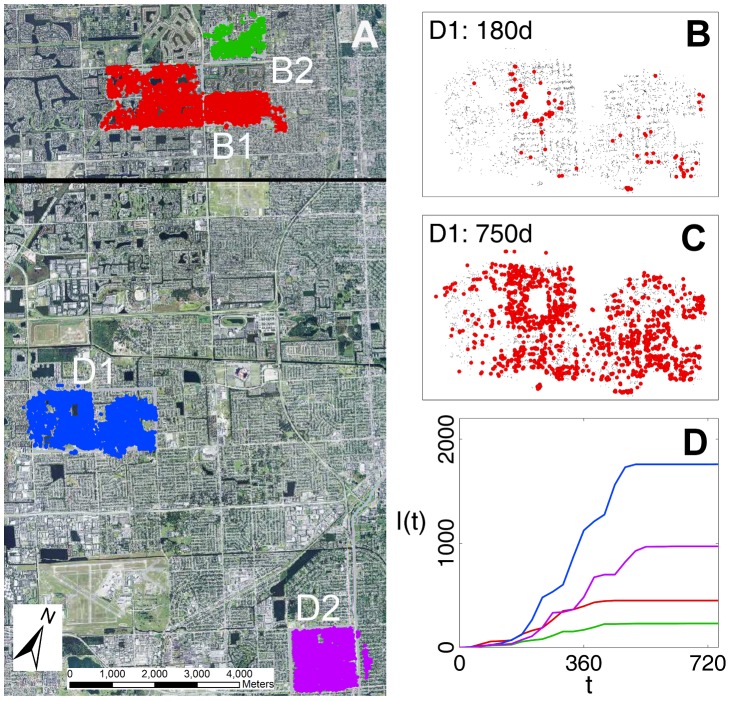
Census sites and progress of citrus canker in urban Miami. **A** Map showing locations and boundaries of four census sites in Broward (B1, B2) and Dade (D1, D2) counties. The coloured outlines indicate the locations of susceptible citrus hosts: there were 4703 susceptible tress in B1, 1113 in B2, 6056 in D1 and 6072 in D2. **B, C** Examples of snapshots at two representative times, 180 d, 750 d in site B2. Grey points indicate locations of healthy (i.e. susceptible trees), red dots indicate locations of newly infected trees within the previous 30 d interval. **D** Increase in numbers of infected trees in successive 30 d intervals at all three sites with colours corresponding to coloured sites in Figure 1A. Background image in Figure 1A provided by W. Luo, courtesy of USDA Service Center Agencies.

The data used for analysis consist of four sites in urban regions close to Miami (Figure 1A), with two sites in Broward County (labelled B1 and B2) and two in Miami Dade county (D1 and D2). The spatial locations of susceptible citrus trees in the four sites were fully enumerated using a differential global positioning system. There were 4730, 1113, 6056 and 6072 trees at sites B1, B2, D1 and D2, respectively. Each site was visited by teams of inspectors at successive intervals between October 1997 and October 1999. The locations of infected trees were identified and notional infection times were calculated by experienced personnel, from lesion size and other phenotypic characters. In order to account for errors in the assessment, the notional times were then grouped into 24 successive 30-day intervals (effectively used as censoring intervals for the true infection times). The data therefore provide spatial snapshots of the locations of susceptible and infected trees at successive 30-day intervals (see examples in Figures 1B,C). The incidence of disease increased rapidly at all sites during the first 18 intervals with little infection thereafter coincident with the onset of dry conditions (Figure 1D). Further details of the collection of data are given in [28]. Disease was present in the area surrounding the census sites during the outbreak, with both susceptible and infected citrus trees between the sites (Figure 1A; see Figure S10 for a density map of citrus trees in the area). The data for an isolated small fifth site, also enumerated by the Agricultural Research Service (ARS) of the USDA, with a very small spread of infection around a single focus of three trees, are not analysed here because the small size of the outbreak precluded rigorous analysis. The effects of ingress of inoculum from infected trees outside the sites were incorporated into the rates for primary infection. Hence, for the purposes of the analyses, in this paper each site was treated as an independent sub-population subject to external inoculum, and parameters were assumed to be independent among sites.

### Models

We consider a family of spatially-explicit, stochastic *SI* models for the spread of disease over time and space through a fixed population of trees (*N*) in each census site. Sites are analysed independently and for notational simplicity the dependence of each parameter on the site is omitted.

#### Infection sources and modes of transmission

The model incorporates two sources of infection: secondary infection by tree to tree spread within census sites, and primary infection from external inoculum coming from outside the site. Secondary infection depends upon the relative locations of infected (*I*) and susceptible (*S*) trees within the site, whereas primary infection depends only upon the availability of susceptible trees. For any pair of infected (*i*) and susceptible (*s*) trees, the probability of secondary, tree-to-tree, infection within a census site depends upon the distance 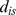 between *i* and *s*, and is given by:
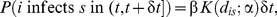
(1)in which 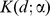 is a dispersal kernel with parameter α, and β is the transmission rate for infection given that inoculum from tree (*i*) arrives at tree (*s*), for a vanishingly small 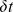, so that no more than one infection event occurs in the interval 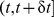.

We extend the generic model to allow for external infection, thus:
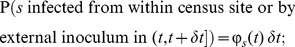
(2a)

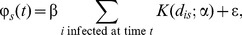
(2b)in which ε is the rate of primary (external) infection per unit time and 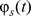 is the hazard, or infectious pressure, for host *s* at time *t*.

Initial inference is focused on three parameters, the primary and secondary transmission rates (*ε* and *β*) and the dispersal parameter (*α*). Later estimation allows for a change in *β* and *ε* over time.

The latent period for citrus canker is short, ∼7–21 days [28] relative to the timescale for infection, and shorter than the interval (30 days) used for data censoring. Hence, latent infection is not represented explicitly in our model. Asymptomatic infection was also not included in the model. The period of asymptomatic infection has been estimated around 100 days [28], which is not negligible compared with the timescale of infection. However, lags in the infection process due to the asymptomatic period were avoided in the analyses described here (see previous section): the dataset used for parameter estimation consists of censored infection times, estimated by pathologists at the time of detection by back calculating from symptom size and expression the likely day of infection with allowance for a 30-day error. See the section “Parameter estimation” below for a test of our assumptions about latent and asymptomatic periods.

#### Spatial dispersal

Here we consider a variety of models: a model with only primary infection (*ε*>0, *β* = 0) in which the infected set at any time is therefore a random selection from the population, as well as spatially-structured models in which we consider dispersal kernels with and without allowance for contemporary external infection. Several different models for dispersal (including the exponential, power law, Gaussian and Cauchy models) were screened for suitability in a preliminary analysis of the data. Two models, with qualitatively different behaviour, fitted substantially better than the others and were selected for comparison: these are the exponential and the Cauchy model, given by:
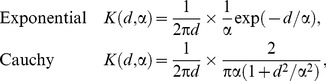
(3)in which *d* is the Euclidean distance between a given pair of infected and susceptible trees, measured in kilometres. Both kernels in Equations 3 are isotropic, of the form 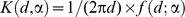, where 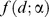 is a one-dimensional kernel defined on the positive real axis (for the kernels in Equations 3, 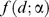 is a negative exponential and half-Cauchy kernel, respectively). A cutoff at short distances was introduced (Text S1, Equations S5) to control kernel divergence. We remark that, owing to the kernel normalisation chosen in Equations 3, the secondary transmission rate *β* is measured in days^−1^km^2^, while the primary transmission rate *ε* is measured in days^−1^ (see Text S1 for a discussion of this point).

The dispersal models differ with respect to the patterns of disease. Whereas exponentially bounded models (such as the exponential) give rise to spreading waves of new infected sites (trees), heavier tailed kernels (such as the Cauchy) result in more dispersed daughter foci ahead of the initial site of infection [33]. The introduction of an external infection rate was supported by the presence of infected hosts around the sites (see also Figure S10 for the population densities), and supplies the system with additional, randomly located primary infections throughout the entire plot.

### Parameter estimation

The transmission (ε, β) and dispersal (α) parameters were estimated by Bayesian inference using Markov chain Monte Carlo methods with data augmentation. Let 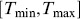 be the time span of experimental observations, *i* a host infected at time 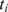 (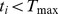), and *s* a host still susceptible at time 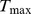. If infection times were known the likelihood function could be calculated as follows:
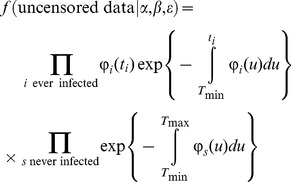
(4)where 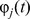 is the infectious pressure for host *j* at time *t* (Equation 2b). However, the data are actually censored, and the likelihood involves integrating over the unobserved infection times consistent with the data: 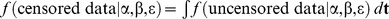. The posterior for 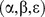 can then be obtained by extending the parameter vector to 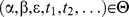, i.e. including the unobserved event times as parameters, and using MCMC to explore the augmented parameter space Θ [34], [35] (for recent applications see e.g. [21], [36]–[38]). The marginal for 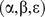 is the desired posterior.

Independent uniform priors over the regions of interest were taken for all parameters, with support coinciding with the following intervals: 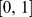
km for α; 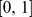
days^−1^km^2^ for *β*; 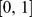
days^−1^ for *ε*. A Metropolis-Hastings algorithm with independent Gaussian proposal distributions [39] was used for parameters α, *β*, *ε*, adjusting the width of the distributions to obtain an acceptance rate between 0.2 and 0.4 for each parameter. The proposal distribution for augmented infection times was constant over the corresponding censoring intervals. Each Monte Carlo chain was run for 100000–250000 steps (depending on the system size and the temporal window used, see below), and a burn-in period corresponding to the initial 10% of the chain was discarded before the analysis, to ensure that convergence had been reached.

Sensitivity analysis was used to test the two assumptions: (i) that the existence of a latent period (∼7–21 days) can be ignored; (ii) that the specific choice of a 30-day censoring interval for true infection times was appropriate given the length of the asymptomatic period (∼100 days). For the first assumption, we compared the fit of the default model with that of a model with a constant latent period (14 days). For the asymptomatic period, we compared the default model with a model fitted to a dataset where the censoring intervals for all infection times were artificially extended to 90 days (with the same midpoints as the original 30-day intervals).

### Model selection

The candidate models were compared for each site separately using the deviance information criterion (DIC, [40]). The objective is to consider whether or not there is evidence for spatially dependent secondary challenge rather than homogeneous primary challenge only, then to distinguish between kernels and whether or not there is evidence for a combination of external (primary) and internal (secondary) infection. The adaptation (DIC_6_) of the DIC suggested in [41] was used to account for the augmented data.

### Estimation using subsets of the temporal snapshots

Following analysis of the entire dataset of 24 successive monthly snapshots of disease, parameters were estimated for subsets of the data in order to identify trends in parameter estimates over time. We also used the analyses to infer what effects additional snapshots or different starting times for data collection would have had on epidemic predictions. For subsequent analyses, we introduce a classification of the models (Table 1) based upon the temporal window used for the estimation (with no reference to the specific form of the dispersal kernel) and the number of parameters used. The original three-parameter model, fitted to the entire dataset, will be denoted with 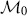. Cumulative windows (model 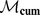 in Table 1) were used to identify the effect of recording more and more snapshots over time on the parameter estimates, by deriving estimates based upon snapshots for 0–3, 0–6, … 0–24 30-day intervals. Sliding windows, for example 0–6, 3–9, …12–18 30-day intervals (model 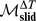 in Table 1, with *ΔT* equal to the window width), were used to assess the effects of different starting times for data collection and fixed periods of observation on parameter estimates (hence, they represent scenarios for later detection and initiation of data collection).

**Table 1:** Main models used in the paper, classified according to the time-dependence of parameters.

Model	Parameters	Number of parameters	Description
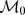	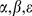	3	All parameters constant; fitted to the entire dataset
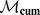	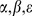	3	All parameters constant; fitted to cumulative time windows of different width, all starting at *t* = 0
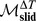	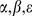	3	All parameters constant; fitted to sliding time windows of width *ΔT* (with different starting times)
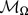	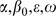	4	Parameters α and ε constant and 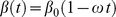 ; fitted to the entire dataset (except for prediction, cf. Figure 6)
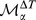	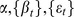	Variable: 1+2×(#intervals)	Parameter α constant, rates 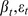 changing by intervals of width *ΔT*; fitted to the entire dataset (except for prediction, cf. Figure 6)

Two additional models were fitted to the entire dataset. Rather than representing scenarios where observation is initiated at different times, as for the sliding-window estimates, these models, like model 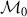, are *post facto* analyses of the epidemic. In a four parameter model, henceforth denoted with 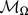 (cf. Table 1), α and ε are constant over time (as in model 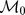), while the secondary transmission rate is a continuous, linearly decreasing function of time, 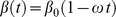, with β_0_ and *ω* constant (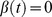 for 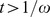). The last model (model 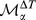 in Table 1) has heterogeneous time scales for the parameters, with α constant for the whole dataset and rates β and ε changing by time intervals. Essentially, this approach implies: choosing a time resolution (e.g., *ΔT* = six months) for the rates 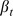 and 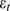; partitioning the whole epidemic time span into regular intervals (e.g., for *ΔT* = 6 months, four intervals: 0–6, 6–12, 12–18, and 18–24 months); fitting different 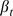 and 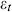 to each time interval (in the same example, four secondary rates 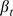 and four primary rates 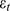), but a single α to all the intervals.

### Comparison of epidemics amongst census sites

We assess the hypothesis that parameters vary spatially between sites as follows. The model is fitted to pairs of sites ***J*** and ***K*** independently (***J***, ***K*** = B1, B2, D1, D2), yielding a sample from the marginal distribution, e.g., for 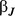 and 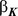 (and similarly for the other parameters) for each of the sites respectively. Under the prior assumption of independence of parameters amongst sites, we can then build a joint posterior distribution for 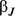 and 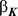, and empirically evaluate the probability 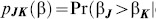
censored data for sites 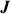 and 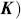. Should 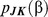 be near 1 or 0, there is evidence that there is a difference in parameter values between sites; if intermediate, the joint posterior straddles the line of equality and we cannot conclude in which location the parameter is greater. Further details are given in [42].

### Goodness-of-fit tests

Goodness-of-fit was tested for parameter estimates from different types of temporal windows using posterior predictive distributions [43]. For each time window (delimited by times 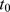 and 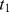, with 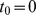 for cumulative windows), a stochastic, spatially explicit model, based upon Equations 2, with parameter values sampled from the posterior distribution, was used to generate a large number (1000) of replicate epidemics, running from time 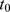 (with initial conditions set according to the recorded infection status) to time 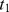. Three summary statistics were stored for each simulation: the count of infected trees, 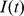, and two spatial statistics, the autocorrelation function 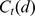 and the “time-lagged” statistic 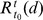, described in detail below. The posterior predictive distributions for stored values of 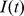, 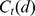, and 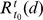 (henceforth, simulated summary statistics), at times *t* corresponding to experimental snapshots, were then compared with the corresponding summary statistics extracted from the experimental dataset (henceforth, experimental summary statistics).

#### Autocorrelation function

We introduce the following definitions: 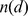 is the number of all tree-tree pairs separated by a distance 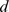 in a given census site; 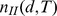 is the number of infected-infected pairs a distance 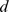 apart at time 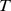 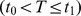; 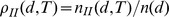 is the corresponding fraction of infected-infected pairs a distance 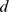 apart at time 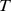; 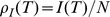 is the fraction of infected hosts at time 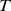. The spatial autocorrelation function at distance 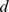 can be defined (see e.g. [44]) as:
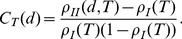
(5)


The non-parametric estimator used here for 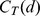 is the *spline correlogram* [45]. A 95% confidence interval for the estimated experimental autocorrelation function was calculated from 1000 bootstrapped datasets, generated from the experimental data, using a dedicated algorithm [45]. Finally, the statistical significance of autocorrelation functions was evaluated by generating 1000 simulated datasets where the infection status of each host was re-allocated randomly (see e.g. [46]). We refer the reader to Text S1 for a brief introduction to spline correlogram calculation and related techniques.

#### Time-lagged spatial statistic

When 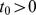, the spatial autocorrelation function between *all* infected trees at time 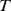 is inevitably offset by the spatial configuration of trees already infected at time 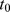, especially in later stages of the epidemic. It is then useful to introduce a statistic that measures the spatial association between “mother foci” (henceforth, *M*), i.e., trees infected at 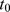, and “daughter foci” (henceforth, *D*), i.e., trees becoming infected after 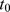. We define 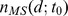 as the number of pairs at time 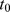 that comprise an infected tree (mother focus) and a susceptible tree a distance *d* apart. At time 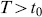, a number 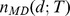 of those initial infected-susceptible pairs have turned into infected-infected (mother-daughter) pairs. If spatial dependence is ignored, the probability for any *M–S* pair at time 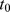 to become an *M–D* pair by time *T* coincides with the probability for an initially susceptible host to be infected between 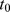 and *T*:
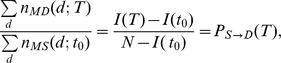
where the sum runs over all existing values of *d*. Under the hypothesis of no spatial dependence between the positions of *M* and *D* trees, the expected value of 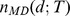 is then given by:
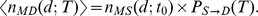



If there is spatial dependence, the probability of observing an *M–D* pair is affected by *d*, and the observed number 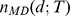 can differ significantly from the expected value. Such difference is measured by the time-lagged statistic 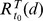:
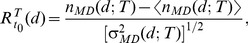
(6)where 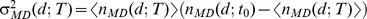 is the sample variance. Deviations of 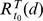 from 0 indicate positive clustering (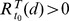) or negative clustering (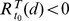). The same techniques described above for spline correlogram estimation were used to obtain smoothed, non-parametric estimates of 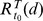, confidence intervals for experimental estimates, and regions of significance (see Text S1 for more details).

### Prediction of epidemic behaviour using estimated parameters

A stochastic, spatially-explicit model, based upon Equations 2, with parameters estimated from different time periods, was used to predict future progress of the disease. Large numbers (1000) of replicate epidemics were generated in each of the census sites, with the susceptible trees located according to the original map for each site and initial conditions set according to the recorded infection status at the time of prediction.

## Results

### Selection of model

A variety of models were compared, comprising secondary infection kernels with and without external infection, and external infection alone. The deviance information criterion (DIC_6_) strongly supported spatially structured models with additional external infection as the most plausible at all four sites. We conclude that, while the epidemic is largely driven by secondary infection between infected and susceptible trees within each site, there are sufficient numbers of isolated new foci at each site to infer that external infection continues to perturb the system. Such disturbance is consistent with long distance dispersal that is known to occur during tropical storms [29], [32].

While DIC_6_ did clearly select for the exponential and Cauchy models with external infection as the most plausible at all sites, amongst all models tested in *post hoc* analysis of the data, it did not give decisive overall support for either (Text S1 and Table S1). The main reason for this, for which we refer the reader to the Discussion and Text S1, is the difficulty in discriminating between long-range dispersal, occurring within a census site, and primary infection incoming from outside. All subsequent analyses apply to the more conservative model with exponential dispersal kernel and external infection. We remark, however, that the results shown below are very similar when using estimates from the Cauchy model with external infection.

Having selected the exponential model from a *post hoc* analysis, we now investigate parameter estimation for this model from early disease snapshots. The kernel type itself could not be identified from early snapshots. Our situation is therefore analogous to a broad class of epidemics in which prior evidence would favour a particular model (here the exponential, or equivalently the Cauchy kernel) and the question is then how soon can the parameters be estimated during an emerging epidemics (see Discussion for further consideration of model selection).

### Sampling windows for parameter estimation of the emerging epidemic

**Figure 2:**
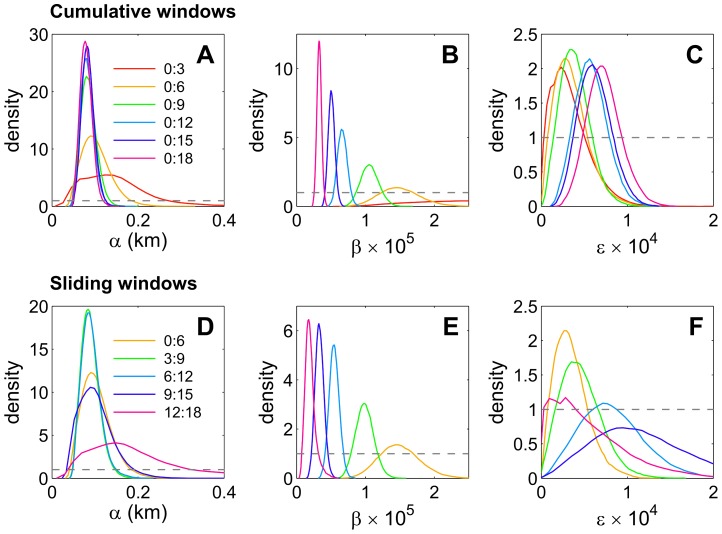
Trend of parameter estimates over time. **A–C** Trends over time in posterior densities for Bayesian MCMC estimation of the parameters *α* (**A**), *β* (**B**) and *ε* (**C**) for a model with an exponential dispersal kernel and external inoculum, based on cumulative windows that successively encompassed 3 additional snapshots of data (model 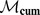, cf. Table 1), extending from 0–3 to 0–24 months. **D–F** Corresponding trends in posterior densities for parameters based on sliding windows encompassing six successive months of observation (model 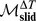 with *ΔT* = 6 months, cf. Table 1), beginning at 0, 3, 6, 9 and 12 months. The figures show marked temporal trends in the transmission rate, *β*, similar temporal trends in the rate of external infection, *ε* and rapid settling of the dispersal parameter, *α*. For each parameter, the gray dashed line represents the prior distribution, rescaled for display by a factor 10^5^ for *β* and 10^4^ for *ε*.

The posterior distributions for the dispersal kernel (α), transmission rate (β), and the ingress of external inoculum (ε) are summarised in Figure 2 for one of the sites (B2) in Broward county. The results show the sensitivity of the posterior distributions of the parameters to the observation time window (cf. Table 1); similar results were obtained for all four sites. Initial inferences were done for cumulative windows (model 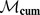, Table 1), in which successively more monthly snapshots of the locations of infected and healthy trees were added. These results show how the availability of additional information during the epidemic affects the precision of the parameter estimates (Figure 2A). The estimate for α is remarkably robust. There is a short, initial transient period (0–3 30-day periods) for which the parameter is not well estimated, by the end of which there are fewer than 21/1113 infected trees. Later estimates were remarkably close both in expectation and precision, with no further gain in precision after six months (Figure 2A), when 69/1113 trees were recorded as infected.

There were clear trends in both the expectation and the precision of estimates for the secondary transmission rate, β. As in the case of α, the posterior distribution for β had a large variance when based upon data for the first three months, and adding extra monthly snapshots decreased the variance of the posterior (cf Figures 2B,E). In contrast with the case of α, there was also a trend in the posteriors for β to decrease as time progressed. The trend in β is more appropriately characterised by the sliding windows (Figure 2E), in which estimates are averaged over successive but overlapping six 30-day intervals (cf. model 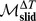 in Table 1, with *ΔT* = 6 months). Similar results were obtained for ε (Figures 2C,F), suggesting that both forms of transmission were driven by environmental variables. Epidemics were dominated by secondary over primary infection: the forces of infection corresponding to β were much greater than those for ε. Hence, in the following we will focus our analysis of environmental trends on the time dependence of β.

The robustness of sliding-window estimates for α to different estimation periods motivates the following assumption: environmental fluctuations affect the model only through primary and secondary infection rates, while the short-range dispersal scale α remains constant at each census site all along the epidemic. We integrated this assumption into our estimations, and fitted to the entire dataset model 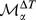, with heterogeneous time scales for the parameters (cf. model Table 1 and Methods), where α was kept constant for the whole epidemic history, while the rates 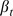 and 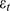 changed with frequency *ΔT*. All the analyses from now on concern model 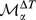, and focus on two different time intervals for the infection rates, obeying two different purposes. The first, *ΔT* = 6 months, is intended to capture the main temporal trend in rates; the second, *ΔT* = 1 month (corresponding to the highest possible resolution given data censoring), is used to analyse short-time fluctuations.

**Figure 3:**
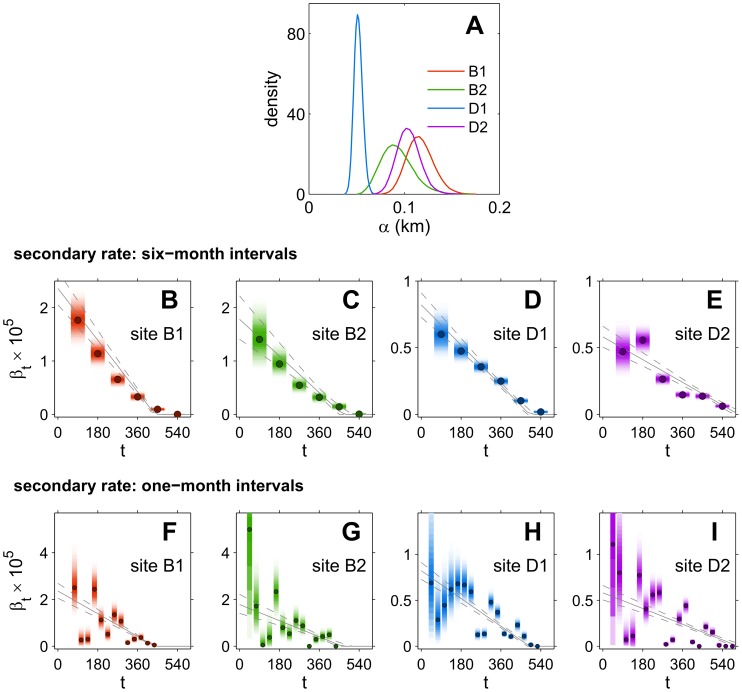
Dispersal scales and trends in infection rates. Parameter estimates for the four census sites, obtained from the model 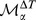 (cf. Table 1). For each site, the value of *α* is constant for the entire epidemic, while the rates *β_t_* and *ε_t_* are time dependent (changing every time interval *ΔT*). **A** Posterior distributions for *α* for the four census sites, from the model with *ΔT* = 6 months. **B–E** Posterior distributions (shaded strips) for *β_t_* for sites B1, B2, D1, D2, from the model with *ΔT* = 6 months. Each strip is centred on the interval used for the estimation, with darker shading corresponding to higher values of the probability density, and dark circles marking the mode of the distribution. **F–I** Posterior distributions (shaded strips) and corresponding modes (dark circles) for *β_t_* for sites B1, B2, D1, D2, from the model with *ΔT* = 1 month. The same conventions as for panels **B–E** are used.

In Figure 3A, we show the posterior distributions for α for the constant-dispersal model 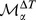 (*ΔT* = 6 months; the posteriors obtained for *ΔT* = 1 month, not shown, are essentially identical). Posterior distributions for the secondary infection rate 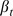 are shown for model 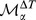 with time resolution *ΔT* = 6 months (Figures 3B–E) and *ΔT* = 1 month (Figures 3F–I). The estimated dispersal length (Figure 3A) is very similar for sites B1, B2, D2, with substantial overlap between the different posteriors for α, and modes ranging between 90 m and 120 m. There is evidence of a shorter mean dispersal length for site D1, with values tightly concentrated around 50 m. Estimates of secondary infection rates 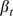 show a decreasing trend common to all four census sites (Figures 3B–I), although with large monthly fluctuations for *ΔT* = 1 month (Figures 3F–I).

The decreasing trend in β can be partly explained by previous investigations [28], which suggested that the epidemic slowed down after ∼12 months because of the onset of an unusually prolonged drought period. Moreover, there is compelling evidence [28] that the three main peaks in the monthly time series for β_t_ (see e.g. months 6, 11, and 15 in Figure 3H for site D1, and similar times for the other three sites) were associated with major rainstorm events (strong wind gusts, combined with rainfall) in the Miami area. For each census site, the decreasing trend is compared (Figures 3B–I, gray lines) with a superimposed trend from the four parameter model 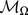 (cf. Table 1 and Methods), in which the secondary transmission rate is replaced by a linearly decreasing function, 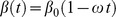, with α, ε, and β_0_ constant (and 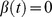 for 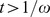). The linear decline in β captures the overall trend, although monthly realisations fluctuate strongly around the trend.

Sensitivity analyses (see Methods) were carried out by fitting model 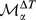 with *ΔT* = 1 month to data for all census sites (cf. Figures 3F–I), either including a latent period of 14 days or by extending the censoring intervals to 90 days. In both cases (results not shown), the choice not to consider latent and asymptomatic period in the models was supported. Estimates with latent period were virtually identical to those in Figures 3F–I; estimates for the asymptomatic period, albeit with some minor deviations, displayed the same pattern as in Figures 3F–I.

### Consistency of parameter estimates amongst different sites

**Figure 4:**
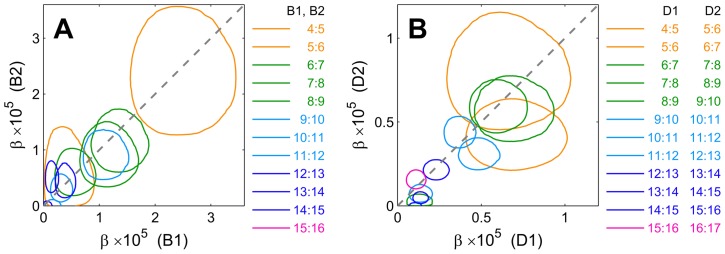
Consistency of secondary rates across sites. Joint posterior distributions for the transmission rate, *β_t_*, for (**A**) the B1 and B2 sites in Broward county and (**B**) the D1 and D2 sites in Dade county. Estimates shown are for the constant-dispersal model 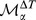, *ΔT* = 1 month (cf. Table 1), discarding an initial transient period of 3 months. Each contour plot corresponds to a value of the probability density equal to 0.05 times the value at the mode. The two Broward sites, which are located close to each other, are similar both in trend and in magnitude of *β_t_*. The two more distant Dade sites show similar trends in *β_t_* once the window for D2 is shifted forward by one month, and a magnitude of *β_t_* approximately 3 times larger in D1 than in D2.

There was evidence of strong consistency for posterior distributions amongst sites. This is shown in Figure 4A, by plotting joint posterior distributions for 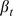 (model 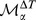, *ΔT* = 1 month, cf. Figures 3F–I) across all four sites. There is a striking correspondence of magnitudes and trends in 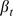 between the two Broward sites (Figure 4A), which are located close to each other (cf. Figure 1A). The more distant Dade sites (Figure 4B) are themselves more distantly separated than the Broward sites (cf. Figure 1A) and show at first a less consistent pattern (see Figure S8). However, if we allow for a 1-month lag in the rates between D1 and D2, the two series of estimates display again a strong correlation (Figure 4B). Such a time lag would be consistent with delayed introduction of the pathogen or the vector, but awaits further analysis and testing. Similar, yet more regular patterns at all four sites emerge when comparing estimates at resolution *ΔT* = 6 months (see Figure S9).

### Goodness-of-fit tests

**Figure 5:**
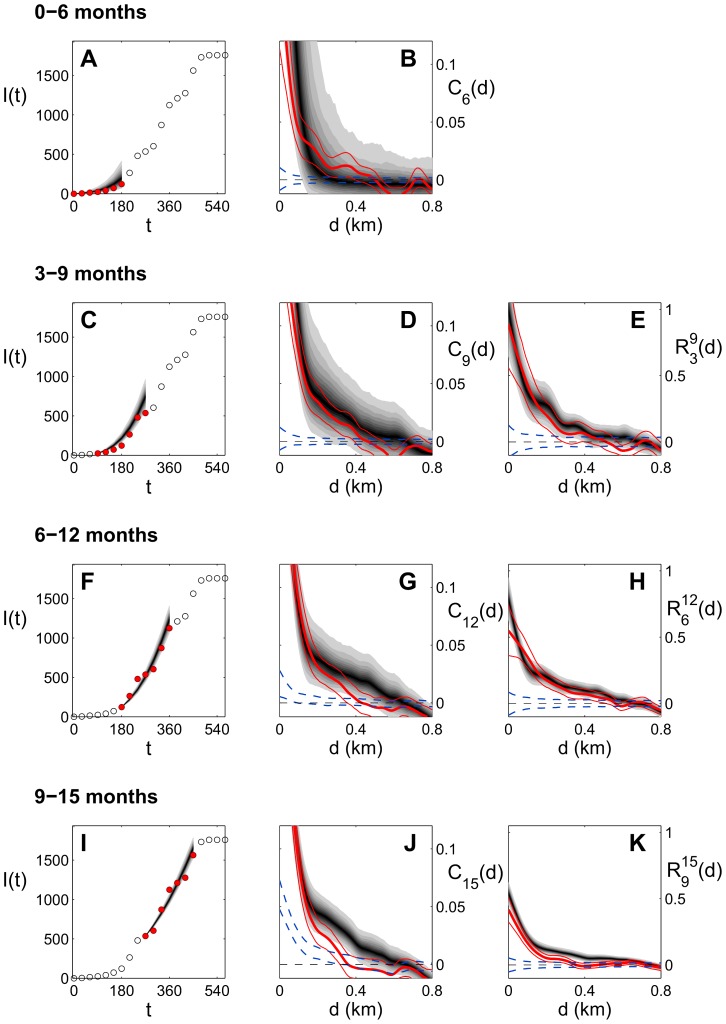
Posterior predictive distributions for the site D1. Results for the constant-dispersal model 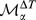, *ΔT* = 6 months (cf. Table 1) are shown for four different intervals (each delimited by times *t*_0_ and *t*_1_, with *t*_1_ = *t*_0_+6 months). Parameter estimates obtained for each interval are used to run the model 1000 times between *t*_0_ and *t*_1_, and summary statistics calculated from the output are compared with the data. **A, C, F, I** Distributions of simulated disease progress between *t*_0_ and *t*_1_ (shaded areas, with black corresponding to the median and different levels of gray to different quantiles) compared to observed disease progress (red circles; empty black circles mark data not used in the comparison). The total number of hosts in site D1 is *N* = 6056. **B, D, G, J** The autocorrelation function at time *t*_1_, 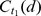, estimated from observed data (thick red line), together with the 95% bootstrapped confidence interval (thin red lines), is compared with the distribution of 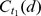 estimated from simulated epidemics (shaded gray, same as for panels A, C, F, I). Dashed cyan lines represent the 95% significance interval found with random labelling techniques. **E, H, K** Time-lagged statistics calculated between times *t*_0_ and *t*_1_, 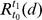. Thick red lines are 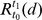 estimated from observed data, thin red lines mark the 95% confidence interval, dashed cyan lines mark the 95% significance intervals, and distributions of 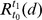 estimated from simulated epidemics are shown in shaded gray.

In Figure 5, we show the results of goodness-of-fit tests for the constant-dispersal model 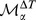, *ΔT* = 6 months (cf. Table 1), for one of the Dade sites (D1; analogous results for the other sites are shown in Figures S2, S3, S4). Intervals 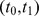 shown are for 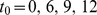 months, with 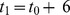 months. Simulated disease progress curves are able to reproduce on average the observed epidemic progress (Figures 5A,C,F,I). The spatial autocorrelation function calculated at the end of each interval, 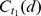 (Equation 5) is shown in Figures 5B,D,G,J. Predictive distributions of 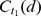 (gray shaded areas) agree well with the autocorrelation estimated from experimental data (thick red lines). Some deviations emerge for the intervals [6], [12] and [9], [15] months (Figures 5G,J), where the experimental function appears to decay faster than the simulated function between 100 m and 250 m (Figure 5G) and 200 m and 600 m (Figure 5J), respectively. The spatial structure of the hosts infected at the beginning of the window (time 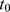) can significantly bias the values of 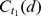: such an effect emerges at short distances in Figure 5J, as the value 0 lies out of the 95% significance interval for 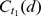 (dashed cyan lines). A statistic free from this bias is the time-lagged function 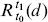 (Equation 6, Figures 5E,H,K), which measures the excess of newly infected trees at time 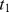 at distance 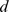 from the trees already infected at 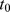. Significance intervals (dashed cyan lines) are always distributed around 0. Predictive distributions of 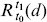 (gray shaded areas) are in very good agreement with 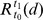 from observational data (thick solid red lines), except again for the interval [9], [15] months (Figure 5K; for a possible origin of the disagreement see Text S1 and Figure S5). Overall, the spatial pattern of the epidemic is broadly well reproduced by the model estimates. We remark (cf. the beginning of this section) that very similar results were found for a model with Cauchy kernel (not shown here). Deviations appear when using different dispersal kernels (considered at the preliminary stage, see Methods), and extreme discrepancies with the data arise when testing models without primary infection (an example is given in Figure S7).

### Predicting the future course of the epidemic

**Figure 6:**
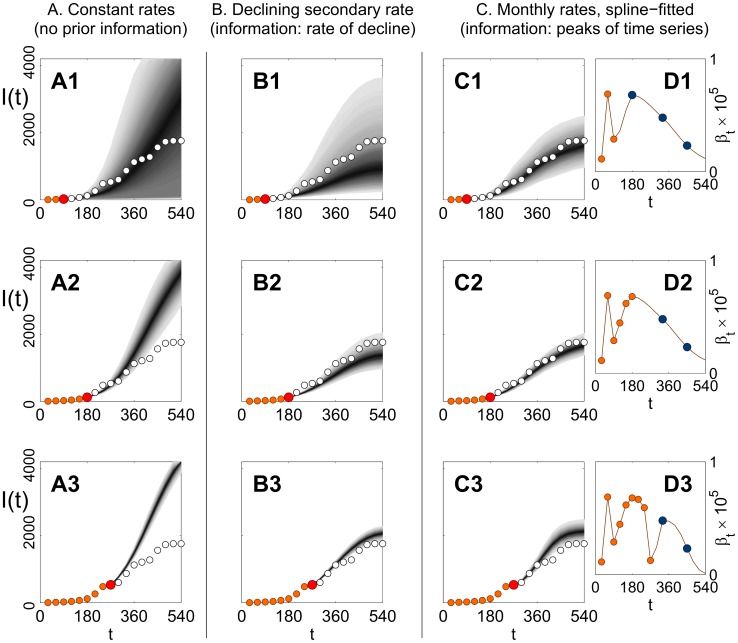
Predictions of epidemic trajectories for site D1. Predictions are based on observation windows of increasing length, comprising data from the first three (**A1**, **B1**, **C1**, **D1**), six (**A2**, **B2**, **C2**, **D2**), and nine (**A3**, **B3**, **C3**, **D3**) snapshots of disease. Three different assumptions (**A**, **B**, **C**) about our prior information on the future evolution of the system were used, each associated to a different model (cf. Table 1). **A1–A3** Predictions based on model 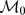, assuming no prior information. The probability distributions for predicted trajectories are shown by gray shading, with intensity of shading representing probability of occurrence. The observational data (disease snapshots) used for prediction are marked by orange circles, the last snapshot used (the prediction time) by a larger red circle, and the observational data to be predicted by white circles. The total number of hosts in the site is *N* = 6056. **B1–B3** Predictions (same conventions as for panels **A1–A3**) based upon model 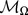, with the assumption that the value of *ω* (the linear decay rate of 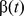, cf. gray line in Figure 3D,H) is known from the beginning. **C1–C3**, **D1–D3** Predictions based upon model 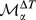 (*ΔT* = 1 month), with constant dispersal parameter *α*, and monthly rates of transmission (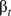, 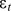) (cf. Figure 3H). **C1–C3** Predicted and observed trajectories (same conventions as in A1–A3). **D1–D3** The associated secondary infection rates 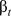, estimated from observed data, marked by orange circles (coinciding with the mode of the distributions; cf. Figure 3H). Predictions are made under the assumption that the positions and values of the peaks in the time series for 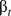 (blue circles in panels **D1–D3**, same as the peaks in Figure 3H) are known in advance. A spline interpolator (dark red line in panels **D1–D3**) is used to impute missing values of 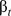.

Strategic decisions about how to react to emerging epidemics are inevitably made early on, when few data are available. However, it is strongly suspected [28] that the main drivers of the epidemic (responsible for the fluctuations and the final slowing down of transmission rates found in our *post hoc* analyses, cf. Figures 3B–I and related discussion) were major weather events that could not be known at the beginning of the outbreak. Such lack of knowledge affects epidemic forecasts made from the early stages of the outbreak. In the following, we investigate three different hypothetical scenarios for early prediction: when no prior information is given about the future conditions of the epidemic (scenario **A**), and when some prior knowledge is assumed (scenarios **B** and **C**). For each scenario, the parameters were estimated using observation windows of increasing size, all starting at *t* = 0, and then used to predict future trajectories of the epidemics up to 18 months (i.e. for the pre-drought period; see above). The results are shown in Figure 6 for one of the Miami Dade sites (D1), with observation windows of 3, 6, and 9 months.

#### Scenario A

(Figures 6A1–A3) The cumulative-window model 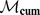 (Table 1) was fitted to the three observation windows. The posterior distributions for *α*, *β* and *ε* were used to generate epidemic trajectories, which were then compared with the true realisation. Predictions based upon initial estimates during the first three months (Figure 6A1) capture the overall trend, although with very wide credible intervals for the ensemble of possible epidemics. As new data for estimation are included (Figures 6A2–A3), the credible intervals tighten, but at the same time the predictions systematically and increasingly overestimate the real epidemic, as they fail in capturing the slowing down of epidemic spread.

As the differences are mainly driven by changes in the transmission rate, β (Figures 3D,H), we tested whether the epidemics could be adequately predicted using model 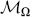, which incorporates a long-term decreasing linear trend: *β*(*t*) = *β*_0_(1*−ωt*) (cf. Table 1). However, the linear trend is confounded by large monthly fluctuations (Figure 3H), and a reliable estimate of the decay rate *ω* was only possible when at least 12 snapshots were used for the estimation (results not shown here). By that time (1 year), the epidemic had already slowed down significantly, and in the circumstances of wanting to predict future disease spread from early observations the estimates would be of little practical use.

#### Scenario B

(Figures 6B1–B3) We investigated whether prior knowledge of the main temporal trend of β (the linear rate *ω*) can improve epidemic forecast. We fitted model 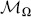 by keeping *ω* fixed to its known value (the mean of the posterior distribution from the “full” estimation, cf. solid gray line in Figures 3D, H), and estimating only α, β_0_, and ε. While very early predictions (Figure 6B1) slightly under-estimate disease (with a very large credible interval), including more snapshots for estimation leads to consistent improvement of the forecast (Figures 6B2,B3). Hence, information about a single parameter, *ω*, leads to a stark improvement of disease prediction. We remark, however, that it was not possible to identify a single, clear environmental factor responsible for the overall decreasing trend of the time series (henceforth, we refer to the *monthly* series only, cf. Figure 3H). Hence, knowing *ω* implies advance knowledge of the behaviour of 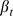 along the whole course of the epidemic. It is desirable to test epidemic predictions under alternative, more parsimonious assumptions about our prior information on 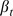.

#### Scenario C

(Figures 6C1–C3, 6D1–D3) We assumed to have prior information about the time of occurrence and values of the three peaks of β_t_ (cf. Figure 3H); no prior information was given about the drought period. We fitted to the observation windows a constant-dispersal model 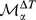 with monthly-varying rates (*ΔT* = 1 month, cf. Figures 3F–I). In Figures 6D1–D3, the modes of the estimated monthly values of β_t_ (orange circles) are shown for each observation window together with the peak values of β_t_ (blue circles) that are known in advance (same values as in Figure 3H). In order to impute the missing values of β_t_, a spline interpolator (dark red line) was built from all the known and estimated values of β_t_. The missing values of ε_t_ were assumed to be constant and equal to the average of ε_t_ over the observation window. Predictions based on the first three months (Figure 6C1, with corresponding estimates for β_t_ in Figure 6D1) capture the future progress of disease, with a smaller credible interval than for scenarios **A** and **B** (cf. Figures 6A1, B1). Increasing the observation window to six and nine snapshots does not have a significant effect on forecast (Figures 6C2–C3), as most of the additional true values of β_t_ (orange circles starting from month 4 in Figures 6D2–D3) are already well imputed from the first three months (cf. corresponding times in Figure 6D1, dark red line). We conclude that knowledge of the peak values of β_t_, supplemented by a few early stage observations, provide enough information to predict the future course of the epidemic. Among the different scenarios we investigated (including several not discussed here), we found scenario **C** to correspond to the *minimal* amount of extra information that could produce reliable predictions from the early stages.

## Discussion

Chief amongst the concerns of policy makers concerned with managing an emerging epidemic are: how far and how fast is the epidemic spreading? How reliable are future predictions of the epidemic severity? Does the epidemic merit the deployment of control, and how should this be optimised? Here we have focused on the first two questions about estimation and prediction, using a combination of Bayesian statistical inference and data for the spread of citrus canker in urban Miami. We assumed that little was known about the pathogen, using non-informative priors for the parameters and a selection of dispersal kernels. Our analyses have shown that the same spatio-temporal, stochastic model is able to capture the temporal trends and spatial statistics characterising the spread of infection in all four sites. Pathogen spread within sites is described by an exponential dispersal kernel with a time-varying transmission rate augmented by a small, time-varying rate of external infection. We show, therefore, that epidemics were not self-contained within sites but new foci of infection also arose from external inoculum, a phenomenon evident at all four sites.

The estimation of dispersal and transmission parameters for stochastic models from spatial snap-shots of disease is not new [21], [47]–[52]. While Gibson and Austin [51] first used likelihood estimation to estimate dispersal parameters from snapshots of citrus tristeza disease in plantations, the current analyses are based upon subsequent MCMC methods to deal with unobserved infection times [34], [35], estimate the most likely chain of infections between successive snapshots [48], [49], and account for temporal variability in transmission parameters [21], [36], [38]. What is different in the current investigation is the quantification of precision and bias of the parameters associated with taking different snapshots of disease over time (Figure 2).

**Figure 7:**
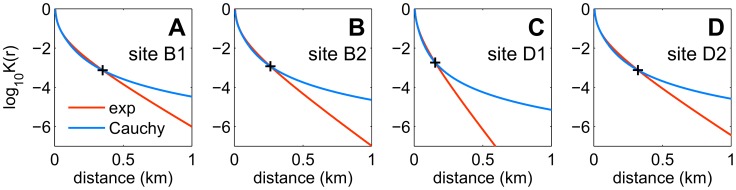
Dispersal kernels as a function of distance. **A–D** Estimated kernels for the exponential model (orange lines) and the Cauchy model (cyan lines), plotted together as a function of distance for each census site. The functional form of the kernels is based upon Equations 3, with a cut-off at very short distances as explained in Text S1 (Equations S5). The mean of the posterior distribution for α for model 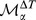 (*ΔT* = 6 months; cf. Figure 3A for the exponential model) is used as a point estimate to plot each kernel. The value of the two kernels at the point where they begin to diverge (“plus” symbol in **A–D**) is about 10^3^ times the value at very short distances.

Models with short-range dispersal (exponential kernel) and long-range dispersal (Cauchy kernel) together with external primary infection were compared using DIC tests (DIC_6_, cf. Table S1 and Text S1). Table S1 shows no significant differences between the exponential and Cauchy models, except for site D1, for which the exponential model is favoured. For the other census sites, the two models are essentially equivalent. This result can be explained in two steps, first by analysing dispersal at short distances (Figure 7), then by considering the contribution of external infection at longer distances (Figure S1). Figure 7 shows a direct comparison of estimated exponential and Cauchy kernels, plotted as a function of distance for each census site. The pattern is qualitatively similar for all census sites: the two kernels are substantially identical up to distances of a few hundred metres (“plus” signs in Figure 7): 250–300 m for all the sites bar D1, and ∼150 m for site D1 (Figure 7C: this may be a reason why the DIC tests favours the exponential kernel for this site). Beyond those distances, which correspond to a fraction of the size of the census site (1 km–4 km), the relative difference between the two kernels increases rapidly. Hence, in principle it should still be possible to detect the effect of such difference in estimates from spatio-temporal maps of disease. However, the long-distance divergence between the two kernels is balanced by the primary infection rate ε. This is shown with an illustrative example in Figure S1 (see also Text S1 for details), where exponential and Cauchy kernels are used to generate spatial maps of the infectious pressure from a given experimental snapshot of site D2 (Figure S1(A)). When only secondary infection is considered, clear differences between the two kernels emerge at long distances (Figures S1(B–C)), but the differences disappear, yielding virtually identical maps, when adding the effect of the external infection rate ε (Figures S1(D–E)). We draw the following conclusion: that the scale of our observations is too small to choose unambiguously between the two dispersal kernels, as the potential effect of long-range dispersal *within* a census site is confounded by the presence of external infection. Gottwald *et al.* [53] found that a power law dispersal model was superior to an exponential model for the spread of ACC in 203 citrus plots in Brazil, following the introduction of the leaf miner. In the absence of the leaf miner, however, dispersal of ACC was adequately described by an exponential model, which is in agreement with our findings; moreover, none of the models considered in [53] included external infection.

We remarked in the results that support for the exponential and Cauchy model was found in *post hoc* analyses of the data. Model comparison from early snapshots supported in general spatially structured models with external infection, but could not select a dispersal kernel (most of the kernels tried, see e.g. Text S1, performed equally well). The choice of an exponential kernel for early estimations (Figure 2) would then be motivated by a strong prior belief on disease dispersal (for example, from results in the absence of the leaf miner in [53]). Here, we also note that, in our case, the absence of such a prior belief would be of little importance, as the exact form of the kernel would not affect the main results of Figure 2. Of the several kernels tried for the first few snapshots, most (e.g. the Gaussian, Text S1) produced estimates of dispersal scale and infection rates with patterns in time qualitatively very similar to those in Figure 2 (results not shown here).

Successful control of disease depends upon matching the scale of control with the inherent spatial and temporal scales of the epidemic [54]–[56]. For our dataset, we have identified a short initial transient period at all four sites for which α and β are not well estimated, with comparatively wider posterior distributions than for later assessments. Clearly, relying upon data for the first three 30-d intervals leads to great uncertainty in estimates of the dispersal scale, and hence decisions about the scale of control (Figure 2). The use of sliding windows shows that fewer but later snapshots could be as precise in estimating dispersal parameters (measured by posterior distributions) as cumulative windows with more snapshots (Figures 2A,D). Estimates for the dispersal parameter changed very little over time (Figure 2D): this motivated consideration of a new, simpler model where dispersal was constant throughout the epidemic (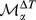, Table 1). The robustness of the results for the dispersal scale was confirmed by goodness of fit tests, in which the posterior predictive distribution of several test statistics showed close concordance with the observed statistics (Figure 5 and Figures S2,S3,S4). The evidence that the dispersal parameter (almost identical for three out of four census sites, Figure 3A) did not change significantly over time, and the fact that this parameter was estimated with substantial precision with few snapshots, are encouraging results in view of control decisions where the scale of control depends on the scale of dispersal [54]–[56].

In contrast with the dispersal parameter, estimates for the transmission rates (β, ε) were not constant (Figures 2E,F and Figures 3B–I), with the secondary transmission rate β showing substantial month to month fluctuations (Figures 3F–H). This result bears two consequences: first, it can frustrate control efforts based on the assumption of a single, intrinsic transmission rate [56]. Second, prediction of future disease severity (upon which the decision to apply control is made) is difficult and prone to systematic error (Figure 6). We suggested that both β and ε were driven by environmental variables that affected the infectivity, and possibly the susceptibility, of the host. Accordingly, we found strong evidence of a time pattern similar among all the census sites for the transmission rate β (Figures 3–4; see also Figures S8, S9). Savill *et al.* [55] explored analogous problems for the infectiousness of infected premises in the 2001 UK foot and mouth epidemic, and identified missing and inaccurate data as a rate-limiting step in refining parameter estimates. For ACC, the principal environmental variables that are likely to influence the pathogen, *Xac*, and the disease are known to be wind-speed, rain and temperature [29], [30], [32]. Extreme weather events have indeed been identified, with robust statistical evidence [28], as the main determinants of the pattern of β (Figures 3F–H): major rainstorm events, acting as environmental pulses, were linked to peaks in the monthly series of transmission rates, and a drought was responsible for the strong quenching of the rates in the second half of the observation period. The existence of a common external driver is also supported (Figure 4 and Figure S9) by the close similarities in the temporal patterns of transmission rates across different sites. Nevertheless, extensive exploratory analysis using environmental data for temperature, wind and rain as covariates did not succeed in identifying a mechanistic environmentally-driven model for β. This was due in part to the (largely unknown) time-lags in the effect of weather events on the pathogen and the host. It is also reasonable to assume that environmental, weather-related forcing was just one, if the most important, of the factors affecting the behaviour of β. Factors intrinsic to the host population might also have played an important role: tree age, cultivar, and horticultural care can affect the susceptibility to the disease [28]. In a population of residential trees, the distribution of such individual factors is extremely heterogeneous in space at several scales, and also fluctuates over time. In the present case, as a result, there was a high degree of spatio-temporal variability in the response of hosts to weather drivers. Fitting models with explicit individual factors is unfeasible in such a highly heterogeneous scenario; however, such a class of models might be very useful in future analyses of outbreaks within commercial citrus plantations, where host properties are more consistently distributed.

We showed that, in retrospect, advance knowledge of major weather events would have been required in order to forecast future epidemic progress. Our methods, based on limited-information forecast scenarios, should be applicable more generally, e.g., to windborne diseases where transmission is mostly driven by strong weather changes. In our analysis (Figure 6), predictions based upon initial estimates, ignoring large weather-related fluctuations in transmission rates (Figures 3E–I), showed progressively more deviation from the actual outcome as more epidemic snapshots were included in the estimation (scenario **A**, Figures 6A1–A3). *Post facto* predictions were effective only when the assumption of complete ignorance of the future was waived (scenarios **B** and **C**, Figures 6B1–B3, 6C1–C6), and some extra information, corresponding to major environmental events, was known in advance (i.e., the drought period and the amplitude of the fluctuations in β in scenario **B**; the peaks of β in scenario **C**). At the same time, of course, meteorological predictability imposes drastic constraints on prior knowledge of that kind. For example, the evolution of position, intensity, and heavier rainfall areas of supercell thunderstorms (two of which were most likely responsible for the first two peaks in the time series for β) can currently not be predicted with more than 2 hours lead time [57]. We can then draw a more general conclusion from our results: that the spatial and temporal scales for prediction must be chosen carefully, not only to match the scales of disease spread [54]–[56], but also with respect to the scales of the weather events that might affect the spread. The spatial and temporal scales considered here (a few km and ∼1 y, respectively) proved to be “too small” for prediction: at those scales, the model output is extremely sensitive to the number and timing of isolated rare weather events (i.e., the effect of those events could not be averaged out). An important question that arises is whether or not our results could be up-scaled: that is, how prediction would perform over larger (e.g., state-wide) spatial scales and longer (e.g., decadal) temporal scales, using the parameter values calculated here and weather templates (cf. [58]) to generate time series for transmission rates. This is the object of ongoing investigation.

Finally, while the lack of predictability is disappointing, it bears an important broader warning, namely that if a component of an epidemic—pathogen, vector or host—is affected by weather, or climate, but that relationship is poorly understood and there are insufficient long-term data, prediction of the future evolution of the epidemic can be both challenging and prone to systematic error. Our system was mainly driven by stochastic weather events occurring on very short time scales. At longer scales, we can consider influenza and mosquito-borne diseases as further contrasting illustrations. Following recent evidence [59] that absolute humidity is a strong driver of the rates of transmission and survival of the influenza virus, a framework to predict seasonal outbreaks of influenza was recently proposed [60]. With daily climatological data and real-time population disease status as inputs, retrospective forecasts could predict historical peaks of influenza outbreak with good accuracy seven weeks in advance [60]. While this case concerns short-term seasonal changes in weather, longer-term changes are also known to influence the risk and spread of disease. The importance of climate on the spread of mosquito-borne diseases is broadly accepted [61]–[63] though very complex and not fully understood [64]–[66]. Large scale weather anomalies, such as unusually long rain [67] or drought periods [68], [69], can lead to unpredictable vector densities, which in turn frustrates public health planning [70]. Global climate change is expected to increase the frequency and intensity of unpredictable extreme weather events, with a far-reaching projected impact on many infectious diseases [70]. In the face of such future challenges, it will be increasingly important for epidemiologists to explore and identify the external factors limiting the predictive capability of their models.

## Supporting Information

Figure S1 **Mapping infectious pressure from primary and secondary sources.** **A** Snapshot of census site D2 at 150 days. The density of susceptible hosts is in gray scale; overlapped red circles are infected hosts. The infectious pressure on susceptible hosts comes from two contributions: secondary sources (red circles) and external sources. **B, C** Infectious pressure from secondary sources only. Maps of the infectious pressure integrated over 30 days (equal to the expected density of new infections), estimated for the **E** model (panel **B**) and for the **C** model (panel **C**). Differences between the two models are evident in the top region of the system, far away from the secondary sources. **D, E** Infectious pressure from primary *and* secondary sources. Maps of the integrated infectious pressure, estimated for the **E** model (panel **D**) and for the **C** model (panel **E**). The differences between the two models disappear when primary infection is taken into account. See Text S1 for a description of the methods used to build the maps and a detailed discussion.
(TIF)

Figure S2
**Posterior predictive distributions for site B1.** Predictive distributions are calculated from estimates for model 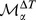, *ΔT* = 6 months (same as Figure 5). Predictive distributions for disease progress (**A, C, F, I**; the total number of hosts being N = 4730), spatial autocorrelation function 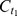 (**B, D, G, J**), and time-lagged statistic 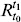 (**E, H, K**) are shown, for intervals (0, 6) months (**A, B**), (3, 9) months (**C, D, E**), (6, 12) months (**F, G, H**), (9, 15) months (**I, J, K**). Symbols and conventions are the same as for Figure 5.(TIF)

Figure S3
**Posterior predictive distributions for site B2.** Predictive distributions are calculated from estimates for model 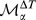, *ΔT* = 6 months (same as Figure 5). Predictive distributions for disease progress (**A, C, F, I**; the total number of hosts being N = 1113), spatial autocorrelation function 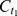 (**B, D, G, J**), and time-lagged statistic 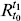 (**E, H, K**) are shown, for intervals (0, 6) months (**A, B**), (3, 9) months (**C, D, E**), (6, 12) months (**F, G, H**), (9, 15) months (**I, J, K**). Symbols and conventions are the same as for Figure 5.(TIF)

Figure S4
**Posterior predictive distributions for site D2.** Predictive distributions are calculated from estimates for model 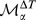, *ΔT* = 6 months (same as Figure 5). Predictive distributions for disease progress (**A, C, F, I**; the total number of hosts being N = 6072), spatial autocorrelation function 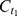 (**B, D, G, J**), and time-lagged statistic 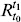 (**E, H, K**) are shown, for intervals (0, 6) months (**A, B**), (3, 9) months (**C, D, E**), (6, 12) months (**F, G, H**), (9, 15) months (**I, J, K**). Symbols and conventions are the same as for Figure 5.(TIF)

Figure S5
**Posterior predictive distributions for site D1: intermediate times.** Autocorrelation 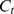 (**A**–**C**; **1**–**3**) and time-lagged statistic 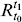 (**A**–**C**; **4**–**6**) (model 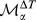, *ΔT* = 6 months, cf. Figure 5) for three time intervals (3–9 months, **A1**–**A6**; 6–12 months, **B1**–**B6**; 9–15 months, **C1**–**C6**), shown at two (**A**–**C**; **1**, **4**), four (**A**–**C**; **2**, **5**), and six months (**A**–**C**; **3**, **6**) from the beginning of each interval. The end-of-interval (six-month) plots are the same as those in Figure 5, while within-interval plots show the evolution of spatial summary statistics. See Text S1 for a discussion.(TIF)

Figure S6
**Posterior predictive distributions for site D2: intermediate times.** Autocorrelation 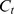 (**A, B, C**) and time-lagged statistic 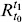 (**D, E, F**) (model 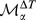, *ΔT* = 6 months) for estimation interval (3, 9) months, at two (**A, D**), four (**B, E**), and six months (**C, F**) from the beginning of the interval. Discrepancies between experimental (red lines) and simulated (grey shaded area) spatial statistics, explained by a lag of the experimental statistics, are solved by artificially shifting forward by two months the experimental autocorrelation function (**J, H**) and the experimental time-lagged statistics (**I, J**). See Text S1 for a detailed explanation.(TIF)

Figure S7
**Posterior predictive distributions from a model with negligible background infection.** Predictive distributions for site D1 are calculated from estimates for model 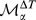, *ΔT* = 6 months (same census site and intervals as in Figure 5), with Cauchy kernel (cf. Text S1, Equation S5b) and background infection *ε* kept at a very small constant value. Predictive distributions for disease progress (**A, C, F, I**; the total number of hosts being N = 6056), spatial autocorrelation function 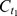 (**B, D, G, J**), and time-lagged statistic 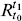 (**E, H, K**) are shown, for intervals (0, 6) months (**A, B**), (3, 9) months (**C, D, E**), (6, 12) months (**F, G, H**), (9, 15) months (**I, J, K**). Symbols and conventions are the same as for Figure 5. For the last three periods (**C–K**), the progress of the epidemic is well reproduced (**C,F,I**), but simulated spatial statistics (**D,G,J** and **E,H,K**) clearly and consistently overestimate experimental spatial statistics (compare with Figure 5, same panels, for the exponential kernel with external infection). See Text S1 for more details.(TIF)

Figure S8
**Temporal pattern of secondary rates in sites D1 and D2: Effect of shift.** Joint posterior distributions for the transmission rate, *β_t_* (model 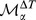, *ΔT* = 1 month) for sites D1 and D2 (cf. Figure 4B), plotted with no artificial shift in time (**A**) and with a 1-month shift in the rates for site D2 (**B**, same as Figure 4B and reproduced here for comparison). While the joint densities in **A** lack a clear correlation pattern, consistency for the two sites emerges in **B** upon introducing a 1-month lag for the parameters of D2.(TIF)

Figure S9
**Consistency of longer-term secondary rates amongst sites: 6-month resolution.** Joint posterior distributions for the transmission rate, *β_t_* (model 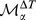, *ΔT* = 6 months; cf. Figure 4 and Figure S8 for *ΔT* = 1 month) for sites B1 and B2 (**A**), sites D1 and D2 plotted with no artificial shift in time (**B**), and sites D1 and D2 with a 1-month shift in the rates for site D2 (**C**). Here, using a lower time resolution for rates, the consistency in the pattern of *β_t_* among census sites emerges with more regularity, although the qualitative behaviour is the same as in Figure 4 and Figure S8.(TIF)

Figure S10 **Estimated distribution of citrus trees in the area of the experiment.** (Figure courtesy of W. Luo.) Area of the Broward County and the Miami Date County surrounding the four census sites (delimited by blue lines, cf. Figure 1A). For each polygon (small sub-areas delimited by gray lines), the human population density and number of households is known from census data. The estimated density of residential citrus trees (colour-coded) was found using an empirical relationship between the number of citrus trees per household and human population density (W. Luo and T. Gottwald, private communication). The estimate shows that the host population was distributed with high spatial heterogeneity around every census site. Moreover, new infections were found in the area, and outside census sites, during all the epidemic (see Methods), which motivates the use of a primary infection rate *ε* in the model (Equation (2b)).
(TIF)

Table S1
**Results of DIC tests.** For each census site, DIC values are calculated for model **E** (exponential kernel and external infection) and model **C** (Cauchy kernel and external infection), with time-dependent infection rates changing by six-month intervals (model 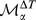 with *ΔT* = 6 months, cf. Figures 3B–E and Table 1) and by one-month intervals (model 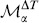 with *ΔT* = 1 month, cf. Figures 3F–I and Table 1). Pairwise differences between DIC values for **E** and **C** models (columns with header **E**–**C**) show that the two models are essentially equivalent, with a trend for **E** to perform better than **C** as the frequency of rate change increases. Only for census site D1 is model **E** clearly favoured. See Text S1 for more details.(PDF)

Text S1 **Dispersal kernels and spatial goodness-of-fit tests: Definitions, basic theory and discussion of further results (including selected supplementary figures).**
(PDF)
