# Supplementary figures and images for: Bayesian Analysis for Inference of an Emerging Epidemic: Citrus Canker in Urban Landscapes
Source: PLoS Comput Biol. 2014 Apr 24;10(4):e1003587. doi: 10.1371/journal.pcbi.1003587 (PMC3998883; doi:10.1371/journal.pcbi.1003587)

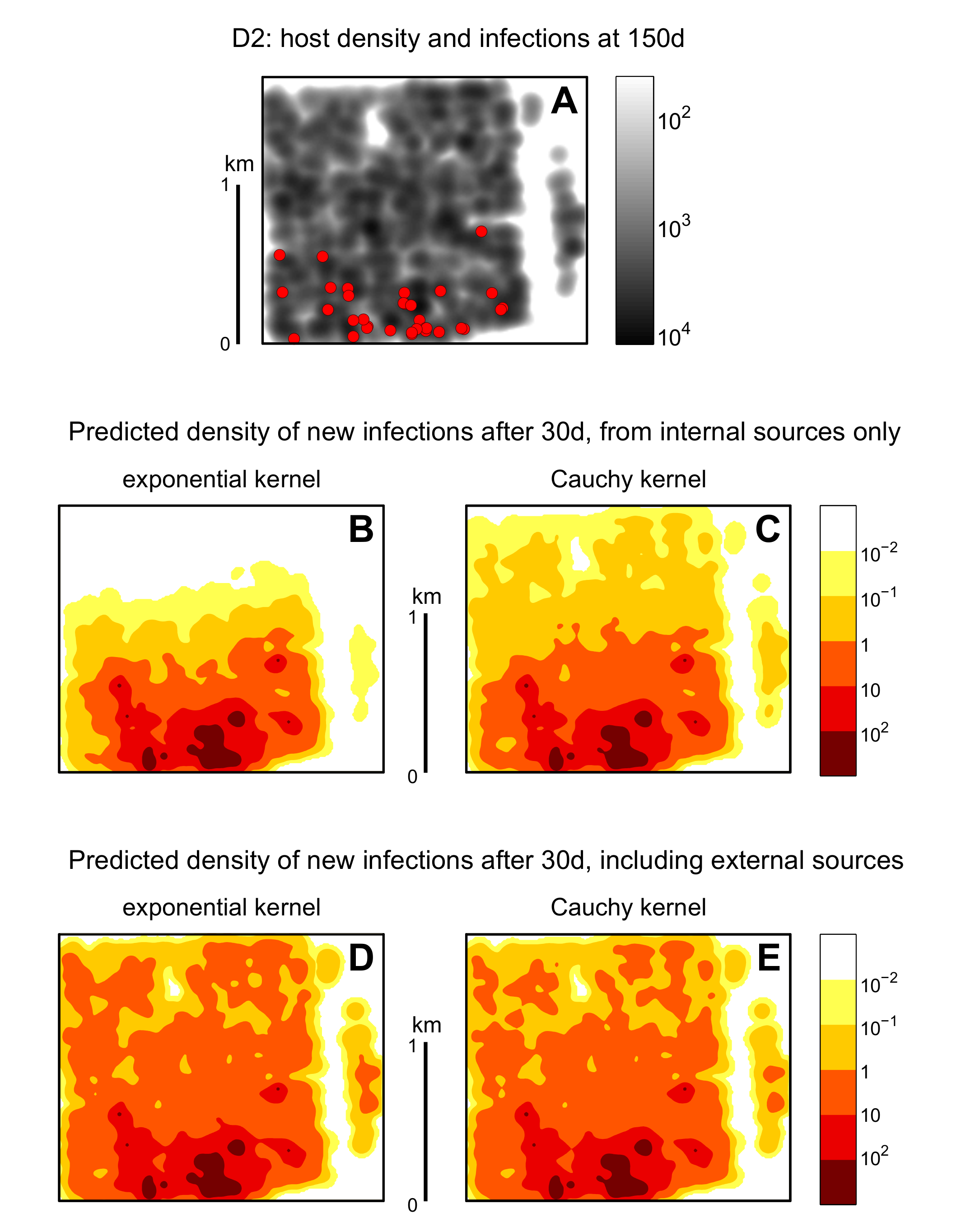

Supplement: Figure S1 — Mapping infectious pressure from primary and secondary sources. A Snapshot of census site D2 at 150 days. The density of susceptible hosts is in gray scale; overlapped red circles are infected hosts. The infectious pressure on susceptible hosts comes from two contributions: secondary sources (red circles) and external sources. B, C Infectious pressure from secondary sources only. Maps of the infectious pressure integrated over 30 days (equal to the expected density of new infections), estimated for the E model (panel B) and for the C model (panel C). Differences between the two models are evident in the top region of the system, far away from the secondary sources. D, E Infectious pressure from primary and secondary sources. Maps of the integrated infectious pressure, estimated for the E model (panel D) and for the C model (panel E). The differences between the two models disappear when primary infection is taken into account. See Text S1 for a description of the methods used to build the maps and a detailed discussion. (TIF) [file pcbi.1003587.s001.tif]

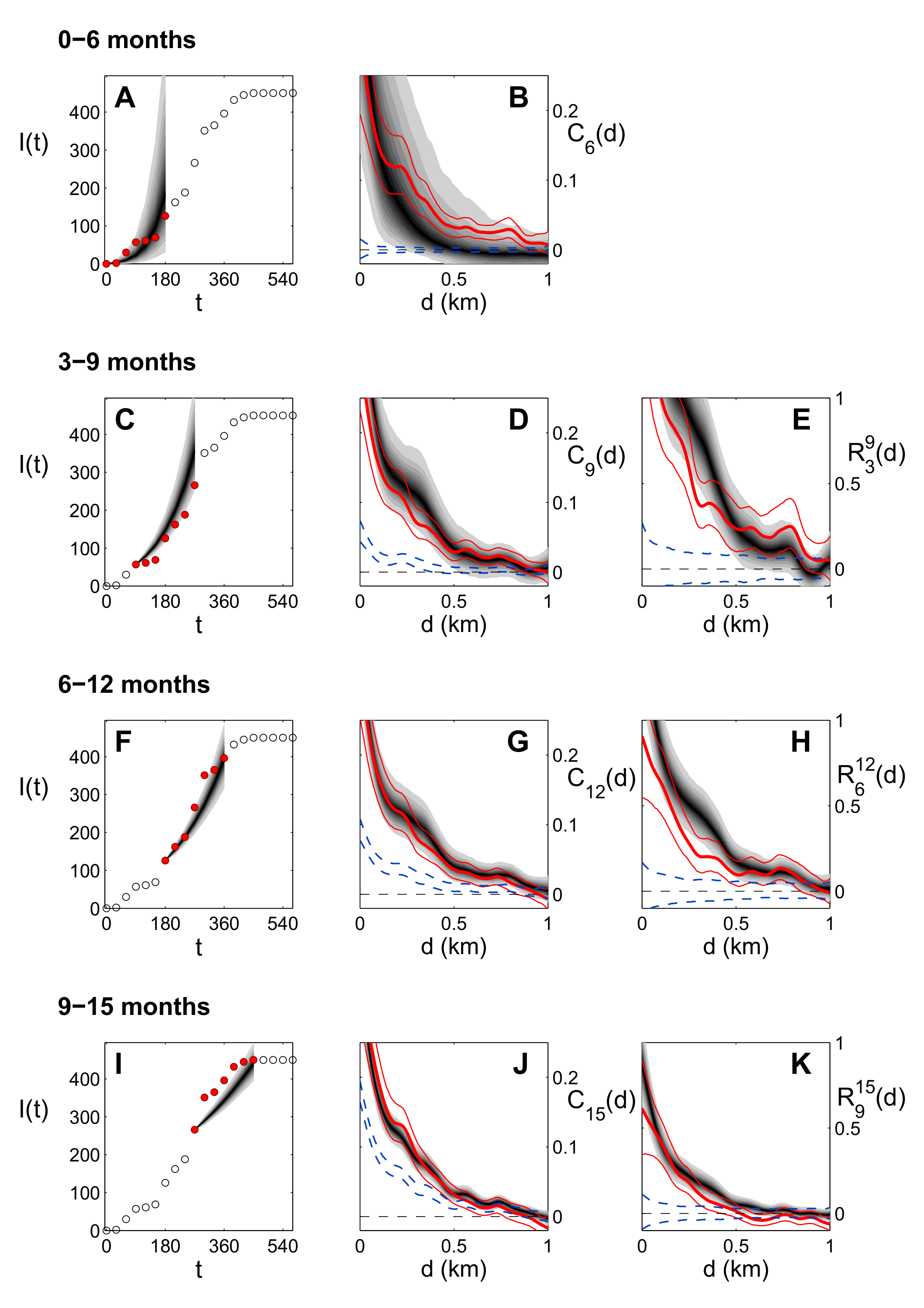

Supplement: Figure S2 — Posterior predictive distributions for site B1. Predictive distributions are calculated from estimates for model , ΔT = 6 months (same as Figure 5). Predictive distributions for disease progress (A, C, F, I; the total number of hosts being N = 4730), spatial autocorrelation function (B, D, G, J), and time-lagged statistic (E, H, K) are shown, for intervals (0, 6) months (A, B), (3, 9) months (C, D, E), (6, 12) months (F, G, H), (9, 15) months (I, J, K). Symbols and conventions are the same as for Figure 5. (TIF) [file pcbi.1003587.s002.tif]

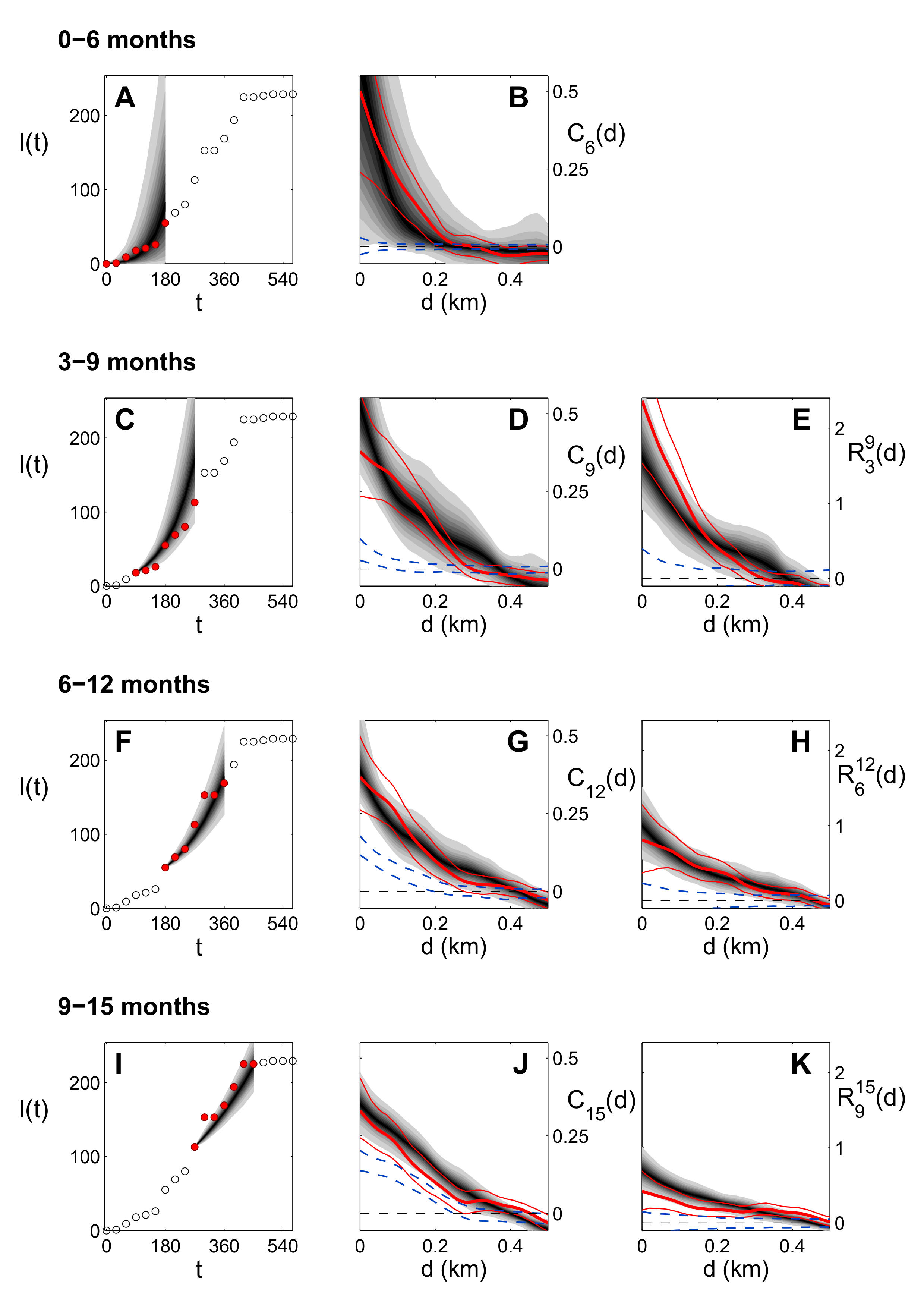

Supplement: Figure S3 — Posterior predictive distributions for site B2. Predictive distributions are calculated from estimates for model , ΔT = 6 months (same as Figure 5). Predictive distributions for disease progress (A, C, F, I; the total number of hosts being N = 1113), spatial autocorrelation function (B, D, G, J), and time-lagged statistic (E, H, K) are shown, for intervals (0, 6) months (A, B), (3, 9) months (C, D, E), (6, 12) months (F, G, H), (9, 15) months (I, J, K). Symbols and conventions are the same as for Figure 5. (TIF) [file pcbi.1003587.s003.tif]

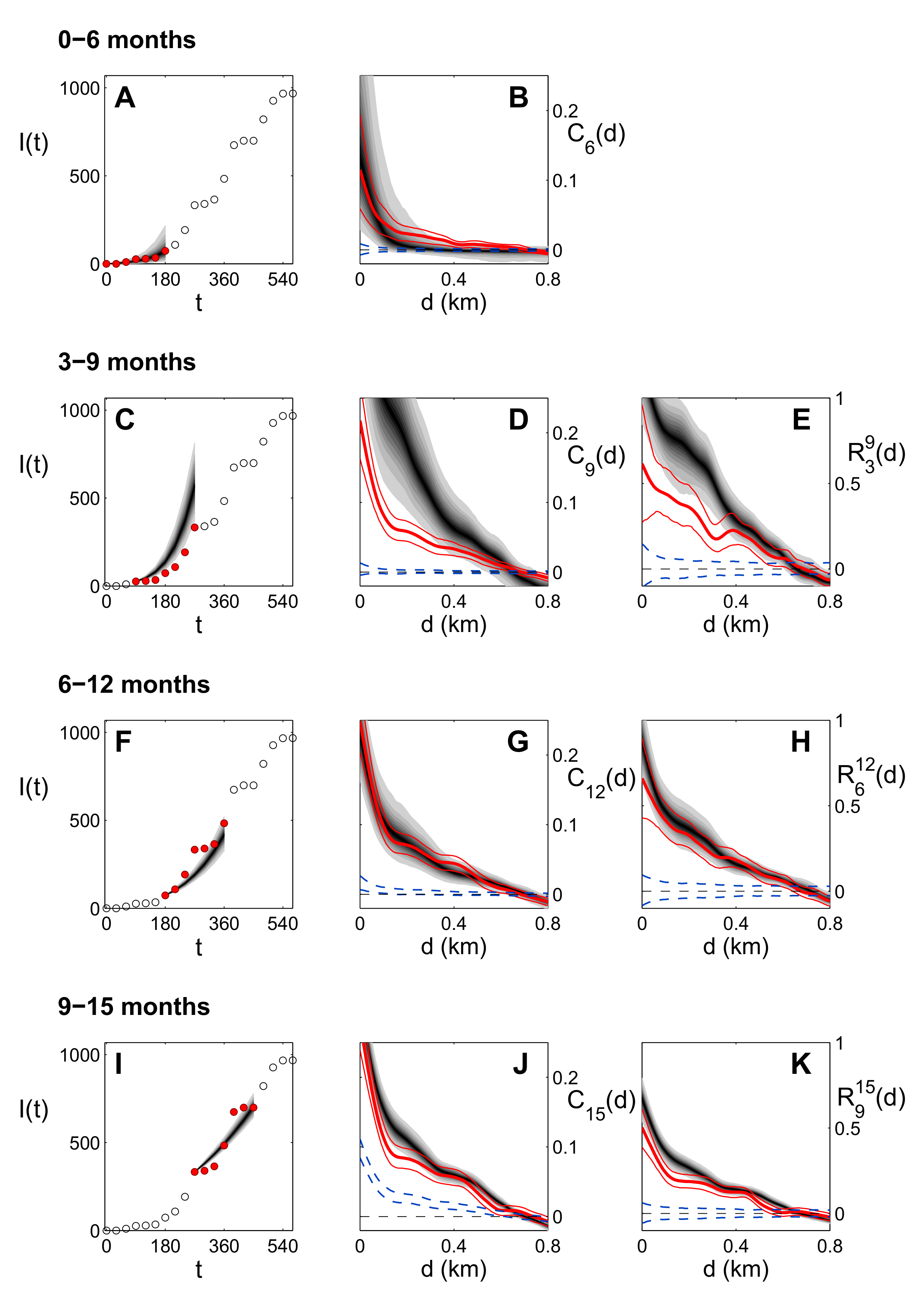

Supplement: Figure S4 — Posterior predictive distributions for site D2. Predictive distributions are calculated from estimates for model , ΔT = 6 months (same as Figure 5). Predictive distributions for disease progress (A, C, F, I; the total number of hosts being N = 6072), spatial autocorrelation function (B, D, G, J), and time-lagged statistic (E, H, K) are shown, for intervals (0, 6) months (A, B), (3, 9) months (C, D, E), (6, 12) months (F, G, H), (9, 15) months (I, J, K). Symbols and conventions are the same as for Figure 5. (TIF) [file pcbi.1003587.s004.tif]

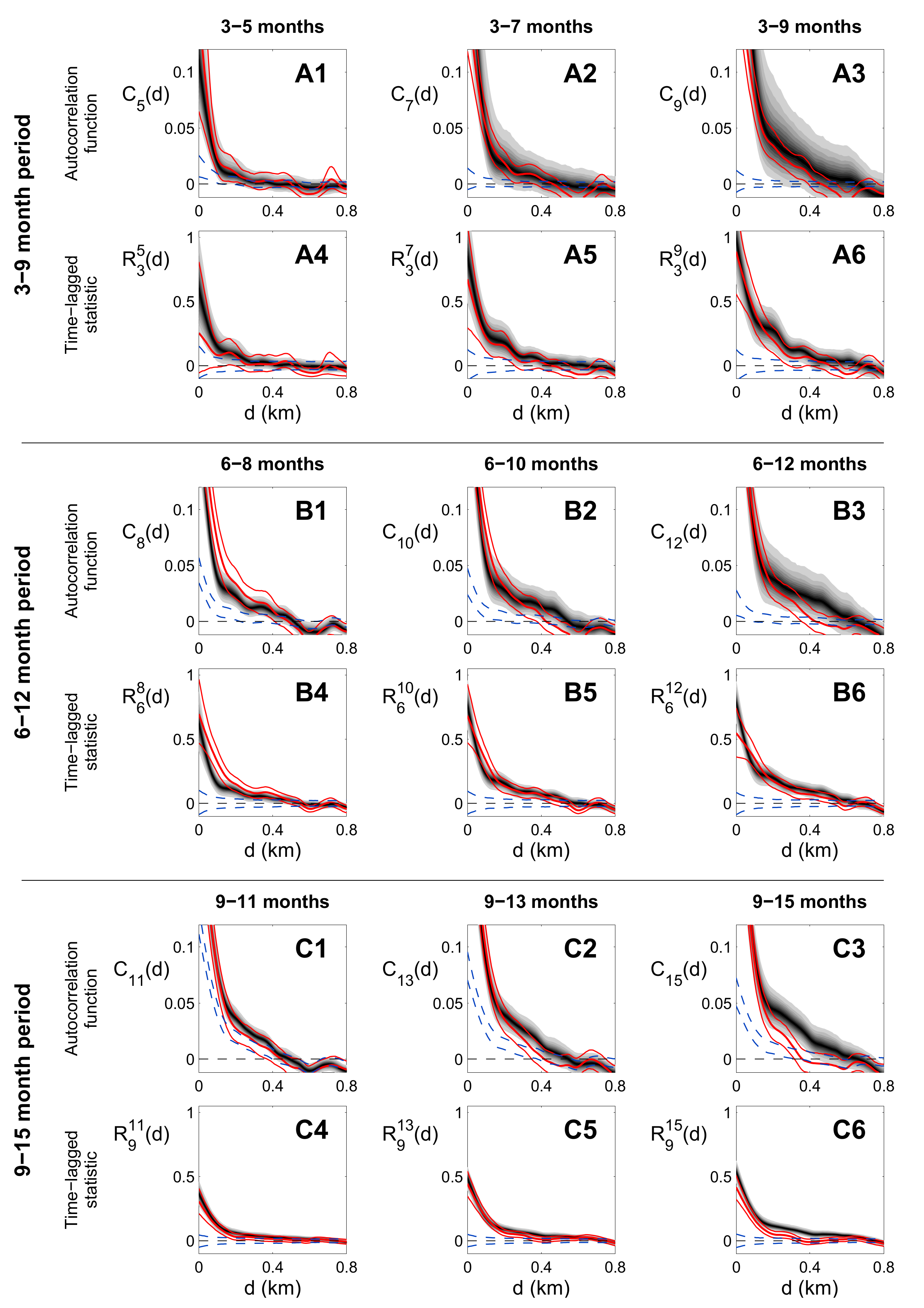

Supplement: Figure S5 — Posterior predictive distributions for site D1: intermediate times. Autocorrelation (A–C; 1–3) and time-lagged statistic (A–C; 4–6) (model , ΔT = 6 months, cf. Figure 5) for three time intervals (3–9 months, A1–A6; 6–12 months, B1–B6; 9–15 months, C1–C6), shown at two (A–C; 1, 4), four (A–C; 2, 5), and six months (A–C; 3, 6) from the beginning of each interval. The end-of-interval (six-month) plots are the same as those in Figure 5, while within-interval plots show the evolution of spatial summary statistics. See Text S1 for a discussion. (TIF) [file pcbi.1003587.s005.tif]

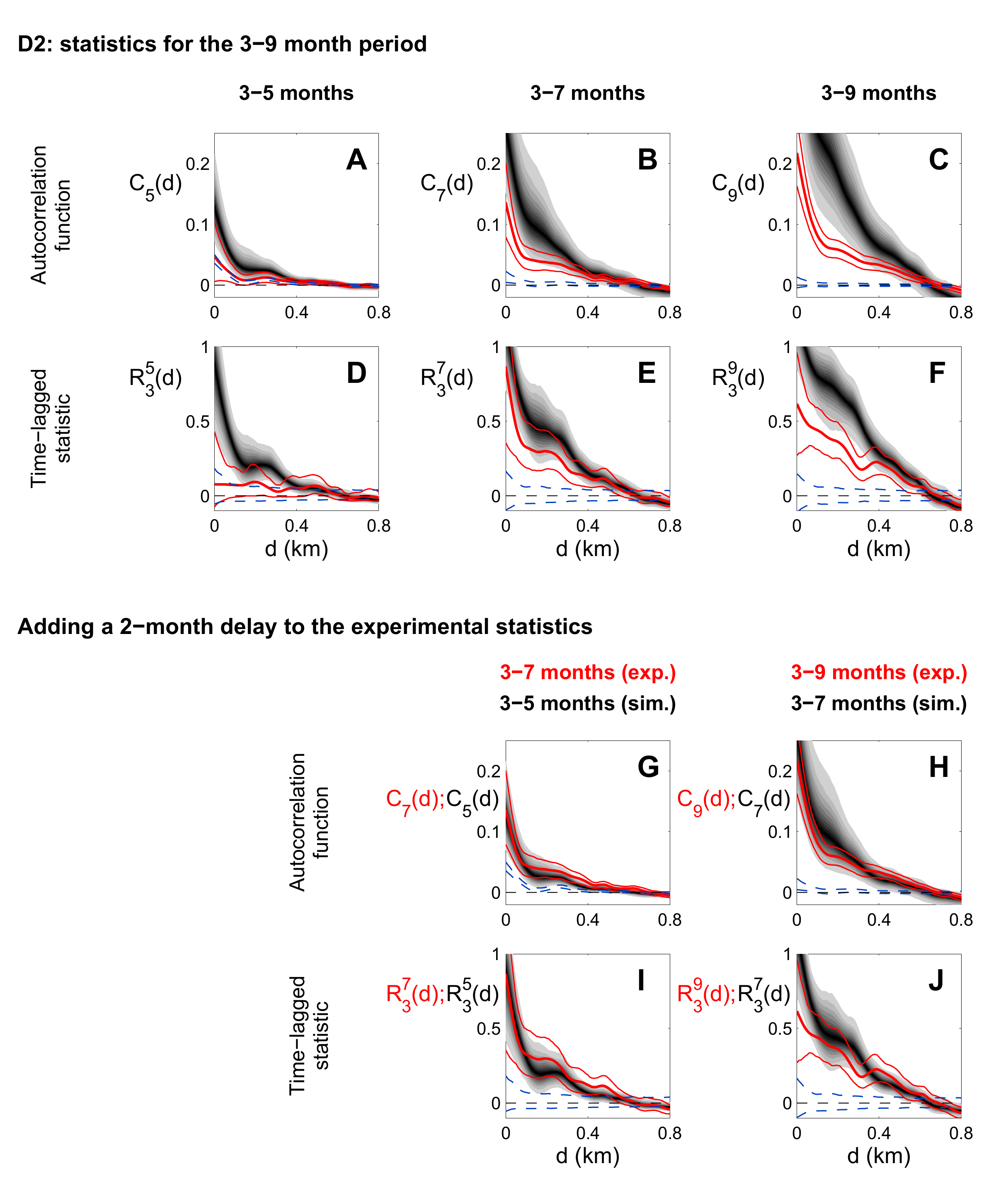

Supplement: Figure S6 — Posterior predictive distributions for site D2: intermediate times. Autocorrelation (A, B, C) and time-lagged statistic (D, E, F) (model , ΔT = 6 months) for estimation interval (3, 9) months, at two (A, D), four (B, E), and six months (C, F) from the beginning of the interval. Discrepancies between experimental (red lines) and simulated (grey shaded area) spatial statistics, explained by a lag of the experimental statistics, are solved by artificially shifting forward by two months the experimental autocorrelation function (J, H) and the experimental time-lagged statistics (I, J). See Text S1 for a detailed explanation. (TIF) [file pcbi.1003587.s006.tif]

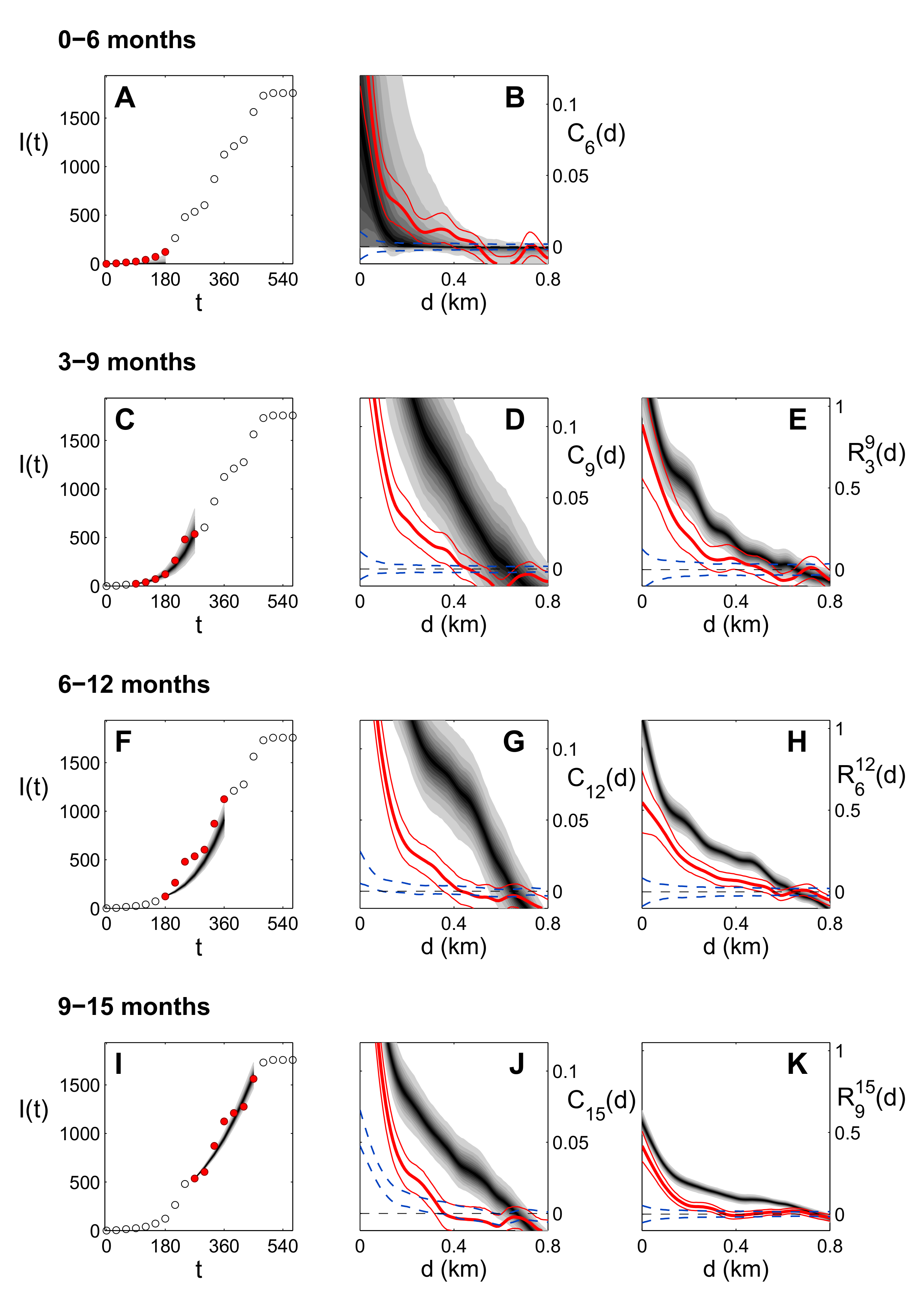

Supplement: Figure S7 — Posterior predictive distributions from a model with negligible background infection. Predictive distributions for site D1 are calculated from estimates for model , ΔT = 6 months (same census site and intervals as in Figure 5), with Cauchy kernel (cf. Text S1, Equation S5b) and background infection ε kept at a very small constant value. Predictive distributions for disease progress (A, C, F, I; the total number of hosts being N = 6056), spatial autocorrelation function (B, D, G, J), and time-lagged statistic (E, H, K) are shown, for intervals (0, 6) months (A, B), (3, 9) months (C, D, E), (6, 12) months (F, G, H), (9, 15) months (I, J, K). Symbols and conventions are the same as for Figure 5. For the last three periods (C–K), the progress of the epidemic is well reproduced (C,F,I), but simulated spatial statistics (D,G,J and E,H,K) clearly and consistently overestimate experimental spatial statistics (compare with Figure 5, same panels, for the exponential kernel with external infection). See Text S1 for more details. (TIF) [file pcbi.1003587.s007.tif]

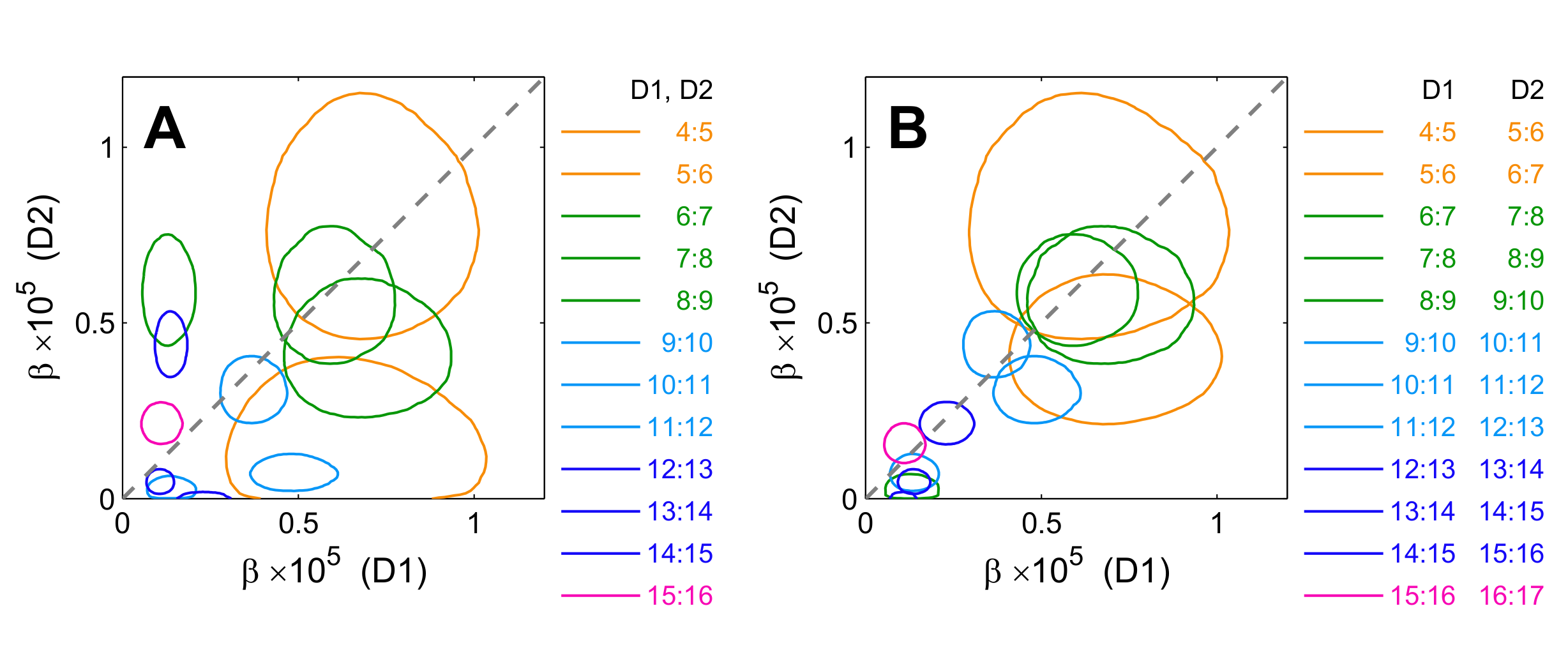

Supplement: Figure S8 — Temporal pattern of secondary rates in sites D1 and D2: Effect of shift. Joint posterior distributions for the transmission rate, βt (model , ΔT = 1 month) for sites D1 and D2 (cf. Figure 4B), plotted with no artificial shift in time (A) and with a 1-month shift in the rates for site D2 (B, same as Figure 4B and reproduced here for comparison). While the joint densities in A lack a clear correlation pattern, consistency for the two sites emerges in B upon introducing a 1-month lag for the parameters of D2. (TIF) [file pcbi.1003587.s008.tif]

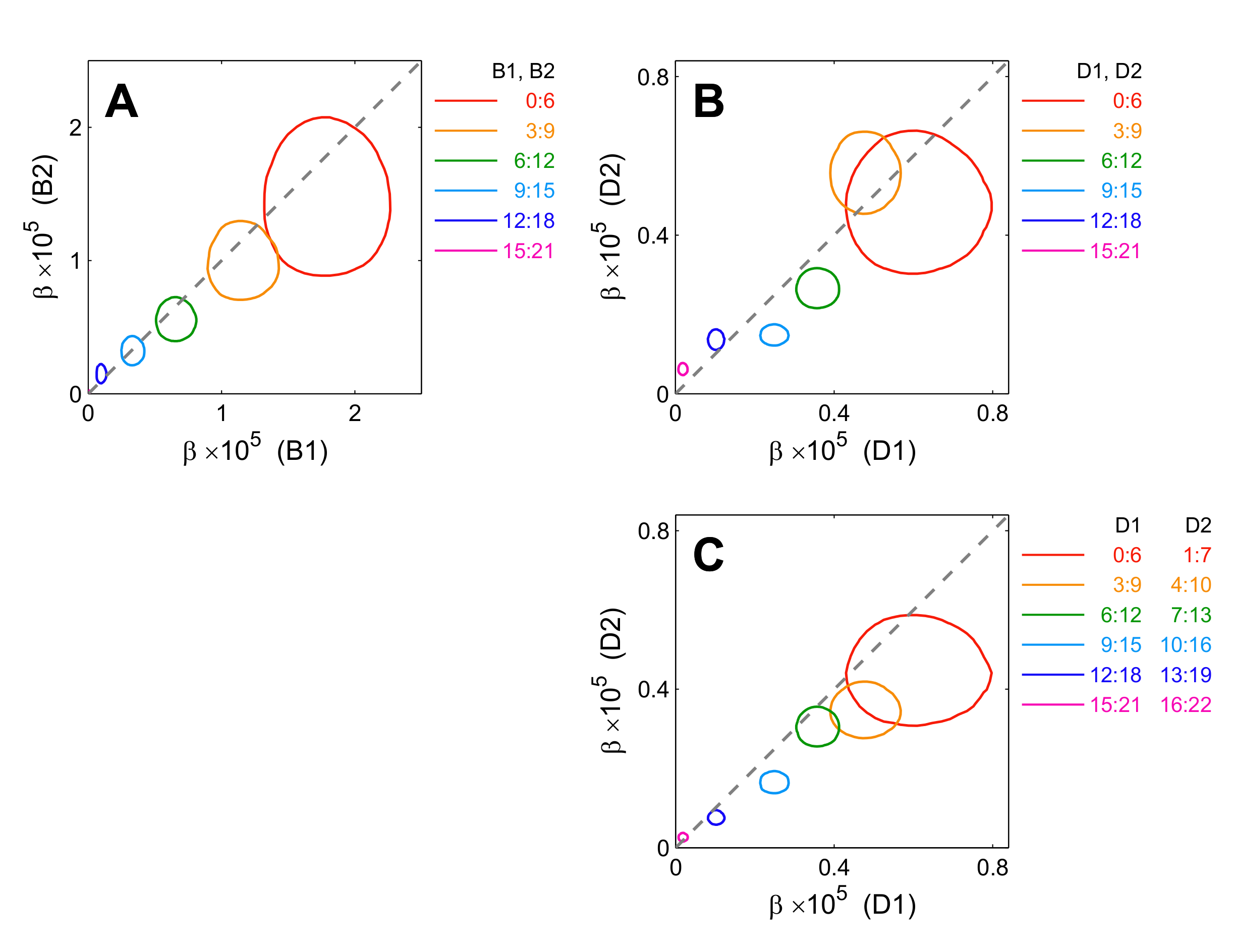

Supplement: Figure S9 — Consistency of longer-term secondary rates amongst sites: 6-month resolution. Joint posterior distributions for the transmission rate, βt (model , ΔT = 6 months; cf. Figure 4 and Figure S8 for ΔT = 1 month) for sites B1 and B2 (A), sites D1 and D2 plotted with no artificial shift in time (B), and sites D1 and D2 with a 1-month shift in the rates for site D2 (C). Here, using a lower time resolution for rates, the consistency in the pattern of βt among census sites emerges with more regularity, although the qualitative behaviour is the same as in Figure 4 and Figure S8. (TIF) [file pcbi.1003587.s009.tif]

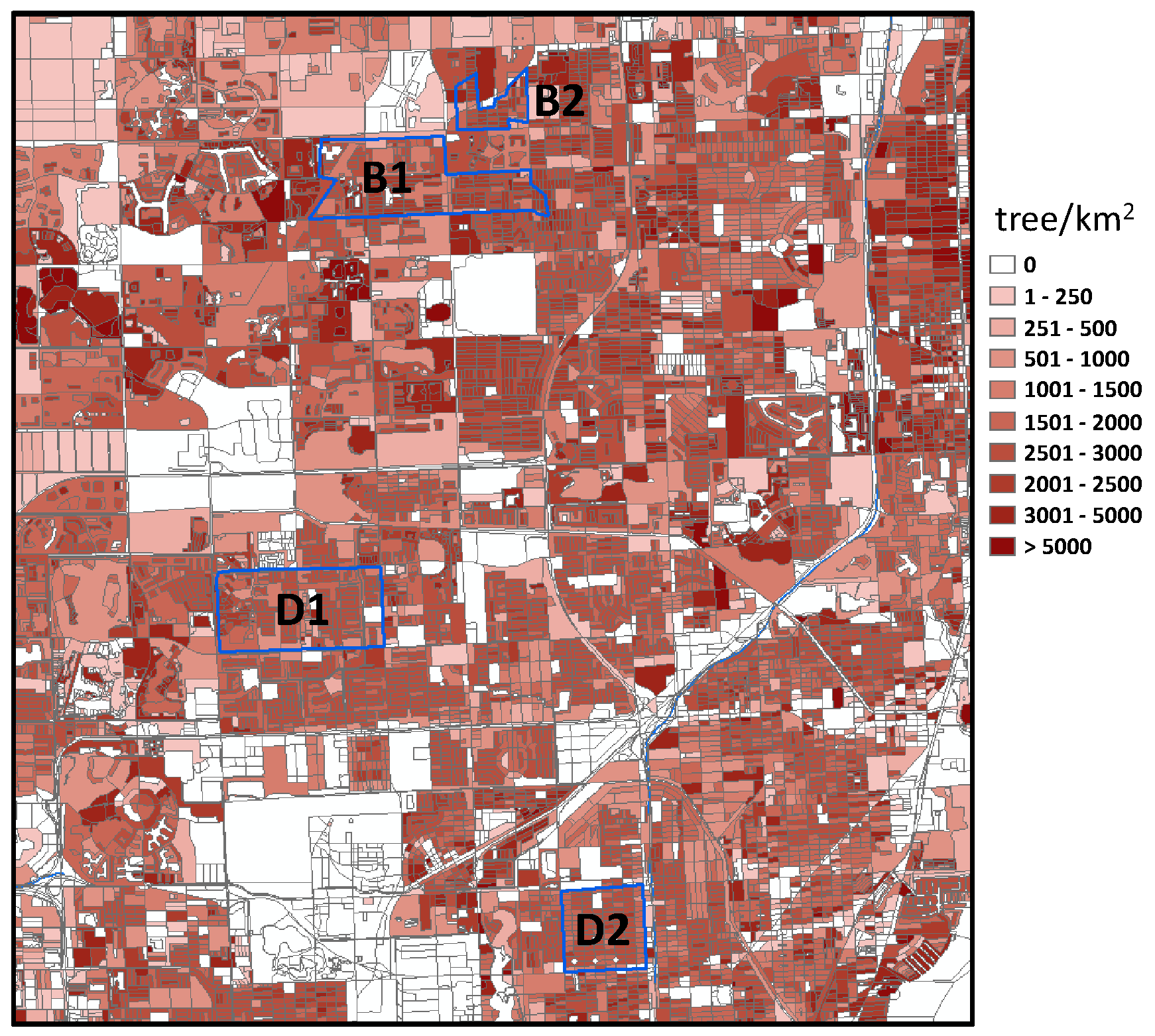

Supplement: Figure S10 — Estimated distribution of citrus trees in the area of the experiment. (Figure courtesy of W. Luo.) Area of the Broward County and the Miami Date County surrounding the four census sites (delimited by blue lines, cf. Figure 1A). For each polygon (small sub-areas delimited by gray lines), the human population density and number of households is known from census data. The estimated density of residential citrus trees (colour-coded) was found using an empirical relationship between the number of citrus trees per household and human population density (W. Luo and T. Gottwald, private communication). The estimate shows that the host population was distributed with high spatial heterogeneity around every census site. Moreover, new infections were found in the area, and outside census sites, during all the epidemic (see Methods), which motivates the use of a primary infection rate ε in the model (Equation (2b)). (TIF) [file pcbi.1003587.s010.tif]
