# Supplementary material for: Bayesian Analysis for Inference of an Emerging Epidemic: Citrus Canker in Urban Landscapes
Source: PLoS Comput Biol. 2014 Apr 24;10(4):e1003587. doi: 10.1371/journal.pcbi.1003587 (PMC3998883; doi:10.1371/journal.pcbi.1003587)
Supplement: Table S1 — Results of DIC tests. For each census site, DIC values are calculated for model E (exponential kernel and external infection) and model C (Cauchy kernel and external infection), with time-dependent infection rates changing by six-month intervals (model with ΔT = 6 months, cf. Figures 3B–E and Table 1) and by one-month intervals (model with ΔT = 1 month, cf. Figures 3F–I and Table 1). Pairwise differences between DIC values for E and C models (columns with header E–C) show that the two models are essentially equivalent, with a trend for E to perform better than C as the frequency of rate change increases. Only for census site D1 is model E clearly favoured. See Text S1 for more details. (PDF) [file pcbi.1003587.s011.pdf]

Table S1: Results of DIC tests.

Census site	DIC for 6-month intervals			DIC for 1-month intervals		
	model **E**	model **C**	**E − C**	model **E**	model **C**	**E − C**
**B1**	7461.2	7461.1	**0.1**	7152.1	7156.8	**−4.7**
**B2**	3619.2	3613.7	**5.5**	3318.4	3318.3	**0.1**
**D1**	27220.9	27236.0	**−15.1**	26376.9	26392.5	**−15.6**
**D2**	16041.5	16041.1	**0.4**	15316.4	15316.8	**−0.4**
