# Supplementary material for: Bayesian Analysis for Inference of an Emerging Epidemic: Citrus Canker in Urban Landscapes
Source: PLoS Comput Biol. 2014 Apr 24;10(4):e1003587. doi: 10.1371/journal.pcbi.1003587 (PMC3998883; doi:10.1371/journal.pcbi.1003587)
Supplement: Text S1 — Dispersal kernels and spatial goodness-of-fit tests: Definitions, basic theory and discussion of further results (including selected supplementary figures). (PDF) [file pcbi.1003587.s012.pdf]

Bayesian analysis for inference of an emerging epidemic: citrus canker in urban landscapes

Franco M. Neri, Alex R. Cook, Gavin J. Gibson, Tim R. Gottwald, and Christopher A. Gilligan

Text S1

Dispersal kernels and spatial goodness-of-fit tests

Contents

1 Dispersal kernels: definition and model comparison **1**

1.1 Dispersal in two dimensions: definition 1

1.2 Short-distance behaviour 2

1.3 Comparison of exponential and Cauchy kernels **(Table S1, Figure S1)** 3

2 Spatial statistics for goodness-of-fit tests **5**

2.1 Spatial statistics 5

2.1.1 Autocorrelation function and spline smoothing 5

2.1.2 Bootstrapping 7

2.1.3 Random Labelling 7

2.2 Further results on spatial goodness-of-fit tests 8

2.2.1 Time evolution of spatial statistics **(Figures S5, S6)**. 8

2.2.2 Spatial statistics to discriminate a wrong model **(Figure S7)** 9

*

1 Dispersal kernels: definition and model comparison

1.1 Dispersal in two dimensions: definition

We assume that inoculum is emitted from a given "source" tree, with emission rate $\tilde{\beta}$, along a random direction given by an angle $\theta \in (0, 2\pi]$ drawn from the probability distribution $g(\theta)$. Inoculum travels along the bearing direction θ and is deposited at a distance r given by the conditional probability distribution, or one-dimensional dispersal kernel with parameter α, $f(r|\theta;\, \alpha)$ (see e.g. [1]). If inoculum dispersal is isotropic, $g(\theta)$ is a uniform distribution, $g(\theta) = 1/(2\pi)$, and $f(r|\theta;\, \alpha) \equiv f(r;\, \alpha)$. The probability that inoculum falls in a small area at distance r and angle θ, delimited in polar coordinates by the diameter dr and the subtending arc $d\theta$ ($dA = r\,dr\,d\theta$), is given by

$$P(dA) \simeq f(r|\theta;\, \alpha)g(\theta)dr\,d\theta = \frac{1}{2\pi}f(r;\, \alpha)\,dr\,d\theta = \frac{1}{2\pi r}f(r;\, \alpha)\,dA. \qquad \text{(S1)}$$

The definition of two-dimensional dispersal kernel, $K(r;\, \alpha)$, is $P(dA) = K(r;\, \alpha)\, dA$. Hence, the relationship between $K(r;\, \alpha)$ and the one-dimensional kernel $f(r;\, \alpha)$ is:

$$K(r;\, \alpha) = \frac{1}{2\pi r} f(r;\, \alpha). \tag{S2}$$

We assume that the probability that inoculum lands on a "target" tree depends on an effective area A_T, unknown but identical for all trees (it could represent, e.g., the projection of the tree crown on the ground, but the specific definition of A_T is not relevant here, as we are not trying to estimate its value). If we assume that A_T is small with respect to any source-target distance r, then the probability that inoculum lands on the target can be approximated by $K(r;\, \alpha)\, A_T$. The rate of infection of the target tree is then given by:

$$\tilde{\beta}\, P_{\mathrm{INF}}\, K(r;\, \alpha)\, A_T \equiv \beta\, K(r;\, \alpha), \tag{S3}$$

where $\tilde{\beta}$ is the rate of emission of inoculum from the source, as above, and P_{INF} is the conditional probability that inoculum infects the target once contact is made. Hence, the infection rate used in the Manuscript is given by $\beta = \tilde{\beta}\, P_{\mathrm{INF}}\, A_T$, with dimensions $\left[\mathrm{L}^{-2}\, \mathrm{T}^{-1}\right]$.

1.2 Short-distance behaviour

We re-write below the dispersal kernels from Equations (3) in the Manuscript, adding the indices E for "exponential", and C for "Cauchy":

$$K_{\mathrm{E}}(d;\, \alpha) = \frac{1}{2\pi d} \times \frac{1}{\alpha} \exp\left(-d/\alpha\right) \tag{S4a}$$

$$K_{\mathrm{C}}(d;\, \alpha) = \frac{1}{2\pi d} \times \frac{2}{\pi\alpha\left(1 + d^2/\alpha^2\right)} \tag{S4b}$$

The information about the spatial location of trees in census sites was obtained using differential GPS (DGPS) modules [2]. The accuracy of the DGPS readings varied between ± 7.5 m and ± 2.0 m, with an accuracy for measured inter-tree distances between ± 15 m and ± 4 m. Kernels (S4a) and (S4b) both have a singularity at $d = 0$, hence they are sensitive to distances below the DGPS accuracy. In order to make our results robust to short-distance observations, we used modified functional forms for the kernels, whereby dispersal coincides with Equation (S4) for

distances d beyond a "hard-core" radius d_0, but is distance-independent for $d \leq d_0$:

$$K_{\mathrm{E}}(d;\,\alpha) = \mathcal{N}_{\mathrm{E}} \times \begin{cases} \dfrac{1}{2\pi\, d_0\, \alpha} \times \exp\left(-d_0/\alpha\right) & \text{if } d \leq d_0, \\ \dfrac{1}{2\pi\, d\, \alpha} \times \exp\left(-d/\alpha\right) & \text{if } d > d_0; \end{cases} \tag{S5a}$$

$$K_{\mathrm{C}}(d;\,\alpha) = \mathcal{N}_{\mathrm{C}} \times \begin{cases} \dfrac{1}{\pi^2\, d_0\, \alpha} \times \dfrac{1}{1 + d_0^2/\alpha^2} & \text{if } d \leq d_0, \\ \dfrac{1}{\pi^2\, d\, \alpha} \times \dfrac{1}{1 + d^2/\alpha^2} & \text{if } d > d_0, \end{cases} \tag{S5b}$$

where $\mathcal{N}_{\mathrm{E}}$, $\mathcal{N}_{\mathrm{C}}$ are normalising constants that can be calculated analytically.

For the sake of completeness, we give below the functional forms of the other two dispersal kernels mentioned in the Manuscript: the Gaussian kernel (with index G) and the power-law kernel (with index P):

$$K_{\mathrm{G}}(d;\,\alpha) = \mathcal{N}_{\mathrm{G}} \times \begin{cases} \dfrac{1}{\pi^{3/2}\, d_0\, \alpha} \times \exp\left(-d_0^2/\alpha^2\right) & \text{if } d \leq d_0, \\ \dfrac{1}{\pi^{3/2}\, d\, \alpha} \times \exp\left(-d^2/\alpha^2\right) & \text{if } d > d_0; \end{cases} \tag{S6a}$$

$$K_{\mathrm{P}}(d;\,\alpha) = \mathcal{N}_{\mathrm{P}} \times \begin{cases} \dfrac{1}{2\pi\, d_0} & \text{if } d \leq d_0, \\ \dfrac{1}{2\pi\, d} \times \left(\dfrac{d}{d_0}\right)^{-\frac{1}{1-\alpha}} & \text{if } d > d_0, \end{cases} \tag{S6b}$$

where again $\mathcal{N}_{\mathrm{G}}$, $\mathcal{N}_{\mathrm{P}}$ are normalising constants. The parameter α has the usual meaning of a dispersal length for the Gaussian kernel (Equation S6a), while for the power-law kernel (Equation S6b) it is a dimensionless parameter with $\alpha \in [0,\,1)$.

Parameter estimates found using different "hard-core" radii d_0 were compared (d_0 ranging from 2 m to 25 m). The results were essentially equivalent in all cases. All the results presented in the Manuscript and in this text were found using the kernels in (S5), with $d_0 = 5\,\mathrm{m}$.

1.3 Comparison of exponential and Cauchy kernels (Table S1, Figure S1)

In Table S1, we report the DIC values for all the four census sites, calculated for the model with exponential dispersal kernel (Equation S5a) and external infection (henceforth, model **E**), and the model with Cauchy dispersal kernel (Equation S5b) and external infection (henceforth, model **C**). We consider models with constant dispersal scale α and time-dependent infection rates β_t and ϵ_t (model $\mathcal{M}_{\alpha}^{\Delta T}$, cf. Manuscript Table 1) varying by 6-month intervals ($\Delta T = 6\,\mathrm{months}$, as in Manuscript Figures 3B-E) and by monthly intervals ($\Delta T = 1\,\mathrm{month}$, as in Manuscript

Figures 3F-I). As a rule of thumb [3], models with DIC within 2 of the "best" (i.e., the lowest score) deserve consideration, and models with a difference greater than 10 are given substantially no support. Table S1 shows a very strong preference for models with monthly-varying rates, which reproduce better the environmental drivers (as expected by comparing the middle and bottom rows in Manuscript Figure 3). It also shows a trend for the performance of model **E** over model **C** to improve going from $\Delta T = 6\,$months to $\Delta T = 1\,$month intervals. The latter effect is most likely due to the time-dependent external rate, ϵ_t, becoming free to vary more frequently and "explain" some infection events that would otherwise require a long-tail kernel. For convenience, the following discussion focuses on the results for $\Delta T = 6\,$months (conclusions drawn from $\Delta T = 1\,$month estimates being very similar).

Table S1 shows no significant differences between the **E** and **C** models, except for site D1, for which model **E** is favoured. For the other census sites, the two models are essentially equivalent. In Manuscript Figure 7, we gave a partial explanation of this result by showing that the dispersal kernels for model **E**, $K_{\mathrm{E}}(d;\,\alpha)$ (Equation S5a) and model **C**, $K_{\mathrm{C}}(d;\,\alpha)$ (Equation S5b) are almost identical up to distances of $\sim$ 300m ($\sim$ 150m for site D1). Beyond those distances, which are still only a fraction of the size of the census sites (1km-4km), the relative differences between **E** and **C** kernels increase rapidly (Manuscript Figure 7). Hence, in principle, it should still be possible to detect the effect of such differences from spatio-temporal maps of disease.

However, the differences between the two kernels are balanced by the primary infection rate, as an illustrative example shows in Figure S1. We consider a disease snapshot, at 150 days, for census site D2 (Figure S1A), and use the observed positions of infected hosts (red circles in Figure S1A), together with the posterior distributions for the model parameters calculated with MCMC, to estimate the spatial distribution of the probability of new infections at the successive snapshot (180 days).

The infectious pressure ϕ_s on a susceptible host s (cf. Equation (2b) in the Manuscript) is given by the sum of two terms: $\phi_s = \phi_s^{(1)} + \phi_s^{(2)}$, where $\phi_s^{(1)} = \epsilon$ is the contribution coming from primary (external) sources, and $\phi_s^{(2)} = \beta \sum_{i\in\mathcal{I}(150\mathrm{d})} K(\|\mathbf{r}_s - \mathbf{r}_i\|;\,\alpha)$ is the contribution from secondary (internal) sources i already infected at 150d. Here, $\mathbf{r}_s \equiv (x_s,\, y_s)$ is the two-dimensional vector of the coordinates of s (the same for $\mathbf{r}_i$), and $\|\mathbf{r}_s - \mathbf{r}_i\| = d_{si}$. The probability that s is infected between 150d and 180d can be estimated as $1-\exp\left(-\int_{150\mathrm{d}}^{180\mathrm{d}} \phi_s(t)dt\right) \simeq \int_{150\mathrm{d}}^{180\mathrm{d}} \phi_s(t)dt = 30\mathrm{d}\cdot\phi_s$, where we assumed that the integral in the exponent is small, and took into account that the infectious pressure $\phi_s(t) \equiv \phi_s$ is constant between 150d and 180d. The reader is warned that the latter assumption was made for the sake of simplicity, and that it neglects the effect on $\phi_s(t)$ of any other host infected between 150d and 180d.

For ease of presentation, we replace the spatially discrete distribution of susceptible hosts with a spatially continuous (smoothed) host density, $\rho(\mathbf{r})$ ($\mathbf{r} \equiv (x,\, y)$), shown in gray scale in Figure S1A (the density units being km^{-2}). Likewise, the infectious pressure on individual hosts is replaced with a spatially continuous density: $\rho(\mathbf{r})\phi(\mathbf{r}) = \rho(\mathbf{r})\phi^{(1)}(\mathbf{r}) + \rho(\mathbf{r})\phi^{(2)}(\mathbf{r})$, where

$\phi^{(1)}(\mathbf{r}) = \epsilon$ as above, while $\phi^{(2)}(\mathbf{r}) = \beta \sum_{i \in \mathcal{I}(150\mathrm{d})} K(\|\mathbf{r} - \mathbf{r}_i\|;\, \alpha)$. Finally, the probability of infection for individual host s is replaced with the expected number of new infections per unit area, $30\mathrm{d} \cdot \rho(\mathbf{r})\phi(\mathbf{r})$. The maps are built using the posterior mean values of the parameters estimated for model **E** (Figure S1(B,D)) and model **C** (Figure S1(C,E)).

In Figure S1(B,C), we show the estimated density of new infections generated by secondary sources, $\rho(\mathbf{r})\phi^{(2)}(\mathbf{r})$. The iso-density curves for the **E** model (Figure S1B) and for the **C** model (Figure S1C) are very similar down to densities of about $1\mathrm{km}^{-2}$ (several hundred times less than the maximum density value). For lower values of the density, given that the internal sources of infection lie in the bottom half of the figure (geographical South), differences between **E** and **C** become visible in the top half (geographical North). In the northernmost region of the census site, the infection density for **C** is still non-negligible, although small, whereas the infection density for the exponential kernel is several orders of magnitude smaller. In the absence of external infection rate, such differences would clearly emerge from parameter estimation (e.g., giving DIC results strongly favouring the Cauchy kernel). However, when the infectious pressure generated by primary sources are added (Figure S1(D,E)), the differences disappear, and maps of potential infections generated by the two models are essentially identical. Hence, exponential and Cauchy models tend to converge in the presence of external infection. The scale of our observations (i.e., the size of the census sites), however, may play a role in this result. It is possible that, with data available at a larger scale, differences between the effect of long-range transmission (both density- and distance-dependent), modelled with a Cauchy kernel, and the effect of primary infection (only density-dependent) would be detectable.

2 Spatial statistics for goodness-of-fit tests

2.1 Spatial statistics

2.1.1 Autocorrelation function and spline smoothing

The estimator of spatial autocorrelation used in the Manuscript is the *spline correlogram* introduced by Bjørnstad and Falck [4]. The main underlying ideas and methods are briefly explained below, following closely reference [4].

We define $z_j(t)$ as the infection indicator for host j at a given time t ($z_j(t) = 0$ if j is susceptible, $z_j(t) = 1$ if j is infected), so that $\bar{z}(t) = \sum_j z_j(t)/N = I(t)/N \equiv \rho_I(t)$ is the average amount of infection in the system at any given time. The sample local autocorrelation between hosts j and k can be estimated as [4, 5]:

$$\hat{c}_{jk}(t) = \frac{(z_j(t) - \rho_I(t))\,(z_k(t) - \rho_I(t))}{\rho_I(t)\,(1 - \rho_I(t))}$$

A widely used, spatially discrete estimator of the autocorrelation function is the *spatial correlogram* [5], which approximates the underlying continuous correlation function with a spatially discrete function. The spatial correlogram is built by specifying a set of distances $\{d_q\}$, $q = 1, \ldots, q_{\max}$, and assigning each inter-point distance d_{jk} to distance class q if it falls within a given "tolerance distance" from d_q: for example, $\delta/2$ being the tolerance distance, a possible choice is $d_q = \delta(q - 1/2)$, with distance class q given by the interval $[d_q - \delta/2,\, d_q + \delta/2)$. The spatial correlogram is defined for d_q as the local average of $\hat{c}_{jk}$ over the associated distance class:

$$\hat{C}(q)_t = \frac{\sum_{j,k=1}^{N} \hat{c}_{jk}(t)\mathbf{1}_q(d_{jk})}{\sum_{j,k=1}^{N} \mathbf{1}_q(d_{jk})}, \tag{S7}$$

where the indicator function $\mathbf{1}_q(d_{jk})$ is equal to 1 if d_{jk} belongs to class q, and equal to 0 otherwise.

The spatial correlogram is the most commonly used estimator of spatial covariance. Some limitations, however, have been remarked [4]. For example, the spatial correlogram is not a valid covariance function itself, as it is not positive definite [6]. Moreover, it is not easy to obtain confidence intervals for the estimator (see the discussion in [4])

Hall and Patil [7] introduced a spatially continuous, kernel-smoothed estimator of the autocorrelation function:

$$\tilde{C}_t(d) = \frac{\sum_{j=1}^{N} \sum_{k=1}^{N} \hat{c}_{jk}(t) K(d_{jk}/h)}{\sum_{j=1}^{N} \sum_{k=1}^{N} K(d_{jk}/h)}, \tag{S8}$$

where $K()$ is a kernel function [8], and the bandwidth h controls the smoothness of the estimator. Equation(S8) gives a consistent estimator of the covariance function, in the sense that it is possible to find a value of h for which $\tilde{C}_t(d)$ converges to the true covariance function (provided that the latter has continuous first and second derivatives) when $N \to \infty$ [6, 7].

In the spline correlogram [4], a cubic B-spline function, $B_\lambda(d)$ [8], fitted to the data, is used as the equivalent kernel smoother (see [9] for seminal work on splines as kernel smoothers, and also [10]). More explicitly, Equation(S8) is replaced with an optimisation problem: finding the function $B_\lambda(d)$ that minimises the *penalised* residual sum of squares:

$$\sum_{j=1}^{N} \sum_{k=1}^{N} \left(\hat{c}_{jk}(t) - B_\lambda(d_{jk})\right)^2 + \lambda \int \left(B_\lambda(d)''(\delta)\right)^2 d(\delta), \tag{S9}$$

where $B_\lambda(d)$ is a piecewise cubic polynomial, and $\lambda \geq 0$ is a smoothing parameter that controls the tradeoff between closeness to the data (first term) and roughness of the curve (second term or "penalty", increasing with the curvature of $B_\lambda(d)$).

The spline correlogram has several desirable properties (see [4] for a discussion): notably, as shown below, it is possible to define a bootstrap method to calculate confidence regions for the estimator. The smoothing parameter λ in Equation(S9) plays a role analogous to the bandwidth h in Equation(S8), i.e., the smoothness of the fit increases with λ. There are several ways to

select the value of λ, hence the degree of smoothness of a spline, for a given problem [8]. A commonly used method is to fix the number of *effective degrees of freedom* df_λ, corresponding to the effective number of parameters used in the fit (see [8] for a rigorous definition). Since df_λ is monotonically decreasing with λ, by choosing df_λ the value of λ is specified automatically.

Spline smoothing is typically carried out with numerical methods [8]. In our case, the spline correlogram routines from a dedicated R package [11], and the related spline fitting routines in the R stats library [12], were adapted and rewritten in C++, in order to be integrated efficiently with our simulation code. In all the results shown in the paper, the degree of smoothing was determined by choosing $\mathrm{df}_\lambda = 15$ for spatial autocorrelation functions estimated in a range of distances from 0 to 1km.

The same smoothing methods were used to estimate the time-lagged statistic, $R^T_{t_0}(d)$. In this case, some of the reasons for choosing the spline correlogram estimator (e.g., positive definiteness) do not apply. The flexibility of splines as kernel smoothers, and the availability of a method to calculate confidence intervals for the estimator, were the main reasons for the choice.

2.1.2 Bootstrapping

We used the dedicated bootstrap algorithm introduced by Bjørnstad and Falck [4] in order to generate confidence intervals for the spatial statistics extracted from experimental data. The status of the system at any given time t is fully described by a set of N triplets (N being the number of hosts in the system): $\{x_j,\, y_j,\, z_j\}$, $j = 1 \ldots N$, where x_j, y_j are the coordinates of host j, and z_j is the infection indicator defined above. A bootstrap dataset was generated by sampling (with replacement) N observations from the original set of triplets. Spatial summary statistics (the autocorrelation function and the time-lagged spatial statistic) were estimated from the bootstrap dataset in the usual way, with the only difference that pairs of the kind j-j, containing two replicates of the same host, were discarded before the computation (to avoid bias towards very small distances). By repeating the process several times (1000), we generated a bootstrap distribution for the smoothed autocorrelation function and the time-lagged statistic for several pre-chosen values of the distance, and used the $2.5 - 97.5\%$ quantile interval at each of those values to build the 95% confidence envelope shown in the Manuscript.

2.1.3 Random Labelling

The significance of spatial statistics calculated both from experimental and simulated datasets was tested against the random labelling hypothesis (see e.g. [13]). A re-labelled dataset can be created by keeping the host locations fixed but re-allocating their infection status randomly: the new set is given by $\{x_j,\, y_j,\, z_{\sigma(j)}\}$, $j = 1 \ldots N$, where $\sigma(j)$ is the j-th element of a random permutation of the indices $\{1, \ldots, N\}$. Smoothed summary statistics can be calculated from the re-labelled dataset in the usual way. A Monte Carlo significance envelope was generated by creating 1000 re-labelled dataset and calculating the 95% confidence envelope from the distri-

butions of summary statistics stored at a set of pre-defined distances. When spatial summary statistics were estimated for intervals (t_0, t_1) with $t_0 > 0$, the significance envelope was found by keeping fixed the infectious status of hosts already infected at t_0, and re-labelling all the other hosts.

2.2 Further results on spatial goodness-of-fit tests

2.2.1 Time evolution of spatial statistics (Figures S5, S6).

In Manuscript Figure 5, we showed only results for the spatial statistics at times t_1 corresponding to the end of the six-month intervals. Statistics calculated at intermediate times can provide complementary, possibly useful information. In Figure S5, we show posterior predictive distributions for site D1, calculated from the same simulation runs as in Manuscript Figure 5, for three different time intervals (3-9 months, cf. Manuscript Figures 5C,E; 6-12 months, cf. Manuscript Figures 5F,H; 9-15 months, cf. Manuscript Figures 5I,K), at 2, 4, and 6 months from the beginning of each interval (statistics at 6 months coinciding with those shown in Manuscript Figure 5). For the 3-9 month period (Figure S5(A1-A6)), and to a lesser extent for the 6-12 month period (Figure S5(B1-B6)), the agreement of simulated and experimental summary statistics is good for all the intermediate times. The simulated autocorrelation functions for the 9-15 month period (Figure S5(C1-C6)) shows good agreement with the experimental autocorrelation functions up to 2 and 4 months from the beginning of the period (Figure S5(C1,C2)), and the discrepancy between simulated and experimental summary statistics observed in Manuscript Figure 5J emerges in the last two months (Figure S5(C3)). The discrepancy is most likely caused by a large above-average fluctuation of the primary infection rate ϵ at month 15 (not shown here), or equivalently, by the fact that a higher-than average fraction of the new infections at month 15 was produced by external sources. Our model (with β and ϵ constant over the 6-month observation period) is not able to capture that effect: as a consequence, the model output contains a higher fraction of infections caused by secondary transmission, which in turn generate higher spatial autocorrelation, as found in our simulated data.

As a second example, we consider Figure S6(C-E) for the spatial statistics of site D2 related to the 3-9 months observation period. The simulated autocorrelation function $C_9(d)$ (Figure S6D) is significantly overestimating the experimental autocorrelation function up to 300m. A similar effect is observed for the time-lagged statistic $R_3^9(d)$ (Figure S6E). Finally, simulated disease progress also consistently overestimates the observed disease progress (Figure S6C). The origin of the discrepancy in the latter case is clear: for two months (the first three red circles from the left in Figure S6C), the observed epidemic spread very little (the cumulative infection count being: 26, 28, 34 infected trees at months 3, 4, 5, respectively). Monthly estimates of the secondary infection rate β are consistently very small for months 4 and 5 (cf. Manuscript Figure 3I). The offset gained by the simulated disease progress in the first two months is maintained up to the end of the observation period (Figure S6C); thus, experimental disease progress “lags behind”

simulated disease progress. The correlation between the positions of infected trees is generated when infection is transmitted from within the system (i.e., via secondary transmission), and increases with the number of sources of infection present in the system at a given time, $I(t)$. Hence, a time lag between simulated and experimental number of infections, $I(t)$, can generate a corresponding time lag between simulated and experimental autocorrelation. In Figure S6, this hypothesis is tested in two stages: first, simulated and experimental spatial statistics are compared (Figure S6(A-F)); second, the experimental spatial statistics is artificially shifted forward in time and the comparison repeated (Figure S6(G-J)). In Figure S6(A-F), we show the statistics at 5 months (A,D), 7 months (B,E), and 9 months (C,F) (respectively 2, 4, and 6 months from the beginning of the period, the latter corresponding to Figure S6(D,E)). At 5 months (Figure S6(A,D)) the simulated statistics (shaded gray) are already significant (as compared with the cyan dash-dotted line), while the experimental statistics (red lines) are not. The discrepancy between experimental and simulated statistics increases at 7 months (Figure S6(B,E)) and 9 months (Figure S6(C,F)). In Figure S6(G-J), experimental statistics at 7 months (Figure S6(G,I)) and 9 months (Figure S6(H,J)) are compared with the corresponding simulated statistics calculated two months before, at 5 and 7 months, respectively. There is substantial agreement between simulated and experimental statistics with the 2-month lag, which confirms that the discrepancy in spatial statistics is mainly due to a delay of two months in the epidemic progress of the experimental system.

2.2.2 Spatial statistics to discriminate a wrong model (Figure S7)

In the Manuscript and in Section 1.3 of the present text we explained that models with exponential dispersal kernel and Cauchy dispersal kernel give substantially identical goodness-of-fit spatial statistics, because of the confounding effect of primary infection. In Figure S7, we show that the spatial summary statistics used in the paper can discriminate between a "good" model (with exponential kernel and primary infection, cf. Manuscript Figure 5) and a wrong model that assumes negligible import of infection from outside (Cauchy kernel with very small primary infection). Model $\mathcal{M}_{\alpha}^{\Delta T}$, $\Delta T = 6\,\text{months}$, with dispersal given by a Cauchy kernel as in Equation (S5b), was fitted to the data for the Miami Dade site D1. The primary infection rate ϵ was not estimated, but kept constant at a small value, $\epsilon = 10^{-6}$ (enough to initiate the epidemic, but negligible for subsequent epidemic dynamics). The estimated parameters were then used to generate simulated epidemic datasets and related summary statistics. The results (for the same time periods as in Manuscript Figure 5) are shown in Figure S7. In the $0 - 6\,\text{month}$ period (Figures S7(A,B)), disease progress is inhibited by the small value of ϵ, hence the results are not relevant to the present discussion. For the other periods (Figures S7(C-K)), although the progress of the epidemic is well reproduced (Figures S7(C,F,I)), simulated spatial statistics (Figure S7(D,G,J) and Figure S7(E,H,K)) clearly and consistently overestimate experimental spatial statistics (compare with Manuscript Figure 5 for the exponential kernel with external infection).

References

[1] Clark J, Silman M, Kern R, Macklin E, Hille Ris Lambers J (1999) Seed dispersal near and far: patterns across temperate and tropical forests. Ecology 80: 1475–1494.

[2] Gottwald T, Sun X, Riley T, Graham J, Ferrandino F, et al. (2002) Geo-referenced spatiotemporal analysis of the urban citrus canker epidemic in florida. Phytopathology 92: 361–377.

[3] Spiegelhalter D, Best N, Carlin B, Van Der Linde A (2002) Bayesian measures of model complexity and fit. Journal of the Royal Statistical Society: Series B (Statistical Methodology) 64: 583–639.

[4] Bjørnstad O, Falck W (2001) Nonparametric spatial covariance functions: estimation and testing. Environmental and Ecological Statistics 8: 53-70.

[5] Cressie N (1993) Statistics for spatial data. Wiley-Blackwell.

[6] Hall P, Fisher N, Hoffmann B (1994) On the nonparametric estimation of covariance functions. The Annals of Statistics 22: 2115–2134.

[7] Hall P, Patil P (1994) Properties of nonparametric estimators of autocovariance for stationary random fields. Probability Theory and Related Fields 99: 399–324.

[8] Friedman J, Hastie T, Tibshirani R (2009) The Elements of Statistical Learning: Data Mining, Inference, and Prediction. Springer Series in Statistics. New York: Springer-Verlag.

[9] Nychka D (1995) Splines as Local Smoothers. The Annals of Statistics 23: 1175–1197.

[10] Hyndman R, Wand M (1997) Nonparametric autocovariance function estimation. Australian Journal of Statistics 39: 313–324.

[11] Bjørnstad O (2012) ncf: spatial nonparametric covariance functions. URL `http://CRAN.R-project.org/package=ncf`. R package version 1.1-4.

[12] R Core Team (2012) R: A Language and Environment for Statistical Computing. R Foundation for Statistical Computing, Vienna, Austria. URL `http://www.R-project.org/`. ISBN 3-900051-07-0.

[13] Cuzick J, Edwards R (1990) Spatial Clustering for Inhomogeneous Populations. Journal of the Royal Statistical Society Series B (Methodological) : 73–104.
